# Supplementary material for: Grid cells are modulated by local head direction
Source: Nat Commun. 2020 Aug 24;11:4228. doi: 10.1038/s41467-020-17500-1 (PMC7445272; doi:10.1038/s41467-020-17500-1)
Supplement: Supplementary file 1 — Supplementary Information [file 41467_2020_17500_MOESM1_ESM.pdf]

Grid cells are modulated by local head direction

Gerlei et al.

Supplementary information

Supplementary Tables

Model	Predicted head direction modulation	References	Notes
Continuous attractor network models	Unidirectional or uniform omnidirectional	[9–11,14]	1
Oscillatory interference models	Uniform omnidirectional	[12,23]	2
Synaptic plasticity models	Uniform omnidirectional	[57,58]	3
Integration of sub-threshold grid fields and location-dependent head direction fields	Localised head direction modulation of grid firing	None	4
Integration of conjunctive cell inputs	Localised head direction modulation of grid firing	Fig. 6 and Fig. S4-6	5

Supplementary Table 1. Comparison of predictions for directional modulation of grid cell firing made by grid models.
References are to example publications and are not exhaustive. Notes for each model:
1. Continuous attractor network models assume that grids result from activity bumps driven around a recurrent network by inputs that encode speed and head direction. Depending on their configuration the models predict either unidirectional head direction modulation (e.g. [59]) or spatially uniform omnidirectional modulation (e.g. [9]). Models with unidirectional head direction firing can account for the properties of conjunctive cells. Because spatially uniform directional modulation is a requirement for these models to generate grid fields they are unlikely to explain the local head direction modulation reported here.
2. Oscillatory interference models generate grid fields through summation (or multiplication) of multiple oscillatory inputs that are tuned to a particular direction and have phase modulated by running speed. Because the effect of direction on firing rate is very weak in these models and as uniform directional modulation of input phase is a requirement for these models to generate grid fields they are unlikely to explain the local head direction modulation reported here.
3. In plasticity models grid fields emerge through synaptic plasticity mechanisms in conjunction with adaptation rules (e.g. [57]) or training signals (e.g. [60]). To date grid cells generated through

these mechanisms appear to have omnidirectional firing fields with no local modulation of directional tuning (cf. [60]).

4. It is conceivable that grid cell firing patterns are generated through a purely sub-threshold mechanism. In other words, grid patterns are generated by membrane potential changes that alone are insufficient to trigger action potentials. In this scenario, additional input from head direction cells with local spatial firing fields, would convert the silent cell into a grid cell with local-directionally modulated firing fields. A challenge for this scenario is to establish biophysical mechanisms that would ensure that alone neither the grid pattern generator, or the head direction input will drive action potential firing.

5. The scenario we proposed here assumes that co-aligned conjunctive cells make convergent synaptic input onto the common postsynaptic neurons. We show here that these postsynaptic neurons will have grid firing fields that are locally modulated by head direction. This model contrasts with the previous scenario (4) which requires multiple relatively complex and untested assumptions.

animal ID	implanted depth (mm)	final location (mm)	distance travelled (mm)	estimated angle	recording site
A	1.6	2.15	0.55	90	deep
B	1.8	2.3	0.5	100	not in MEC
C	1.4	2	0.6	90	not in MEC
D	1.6	2.3	0.7	100	parasubiculum
E	1.5	2	0.5	90	parasubiculum
**F**	**1.6**	**2.3**	**0.7**	**100**	**superficial**
**G**	**1.5**	**2.3**	**0.8**	**90**	**deep**
H	1.5	1.7	0.2	110	superficial
**I**	**1.5**	**2.3**	**0.8**	**100**	**superficial**
J	1.5	2.3	0.8	90	not in MEC
**K**	**1.5**	**2.3**	**0.8**	**90**	**deep**
**L**	**1.5**	**2.15**	**0.65**	**90**	**superficial**
**M**	**1.5**	**2.2**	**0.7**	**90**	**superficial**
**N**	**1.5**	**2**	**0.5**	**80**	**deep**

Supplementary Table 2. Estimated position of tips of recording electrodes at the beginning and end of experiments and estimated recording sites in deep and superficial layers of the MEC. Estimated angles are relative to the straightened skull and are based on histology images. Two animals were terminated for health reasons and their brains were not processed. Animals with grid cells used in the analyses are highlighted in bold.

Supplementary Figures

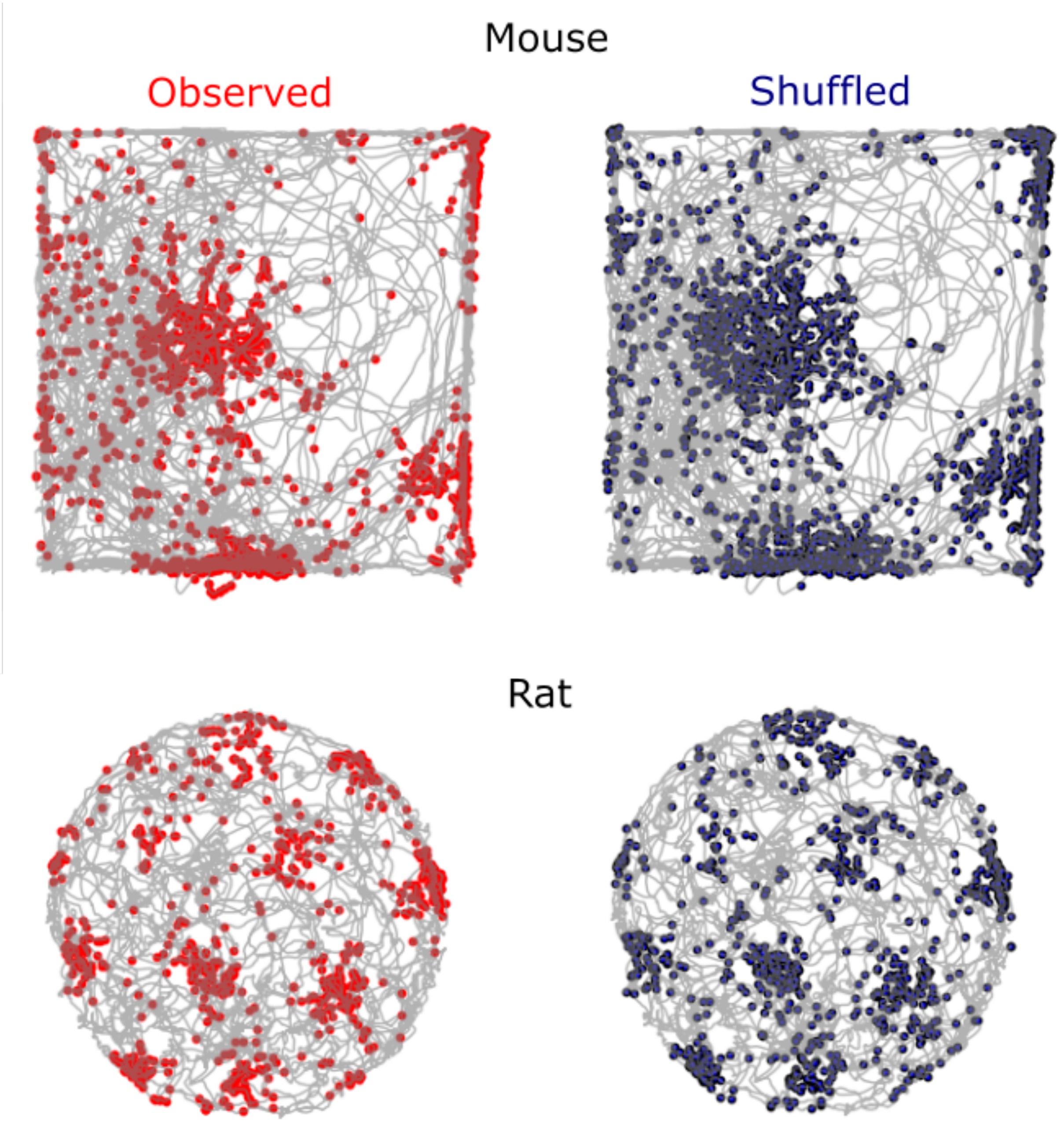

Supplementary Figure 1. Shuffling maintains the location-dependent firing rate. Examples of observed (left) and shuffled (right) spikes from a mouse (top) and a rat (bottom). Grey lines are the trajectories of the animal.

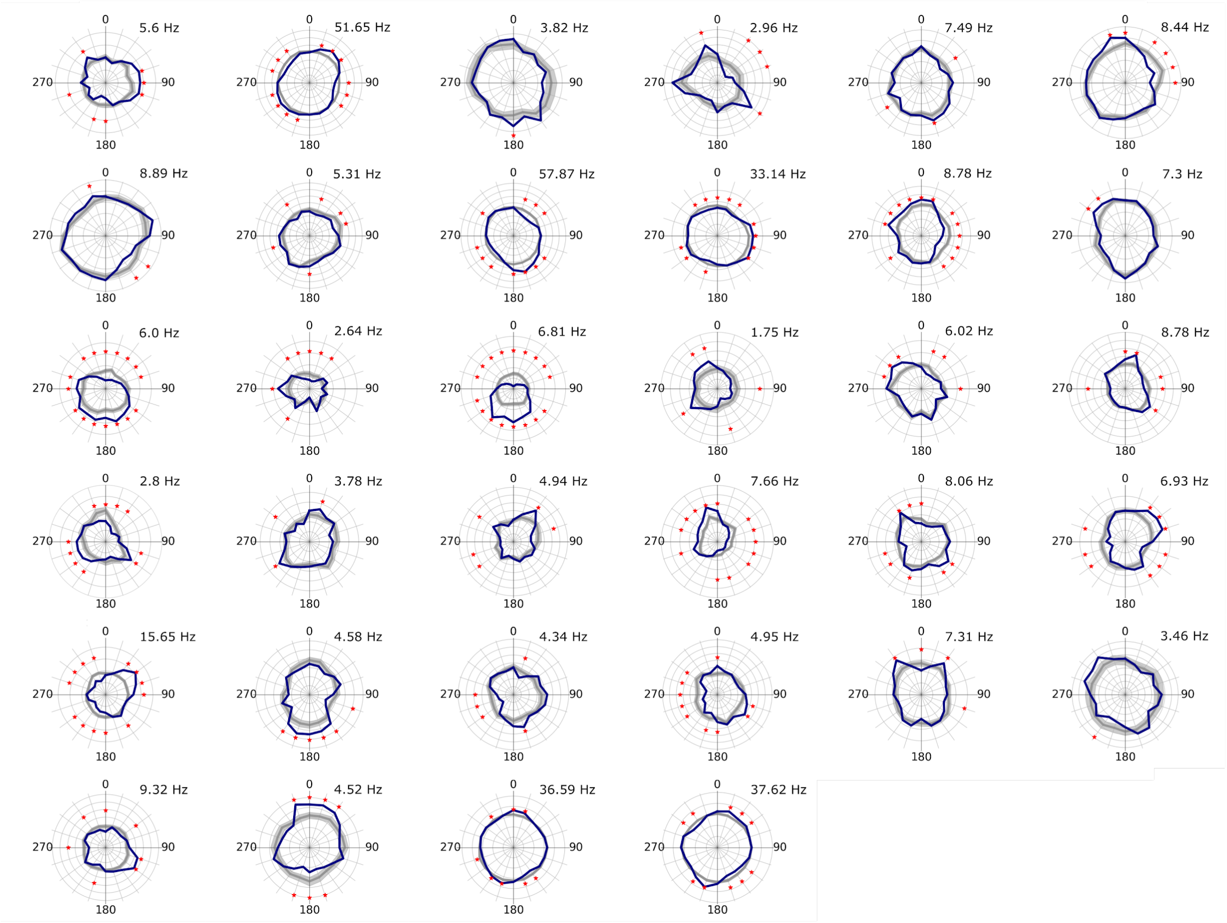

Supplementary Figure 2. Direction dependence of global firing of pure grid cells recorded from mice. The observed firing rate is shown binned as a function of head direction (blue) along with the corresponding shuffled data (grey) for each pure grid cell. The lighter grey region indicates the range of the 5th and 95th percentiles. The maximum firing rate of the observed data is shown above each plot. Significantly directional bars are marked with an asterix (*). Significantly directional bars are where the observed data differs significantly from the shuffled data ($p < 0.05$, two-tailed p value calculated from the shuffled distribution and corrected for multiple comparisons with the Benjamini-Hochberg procedure).

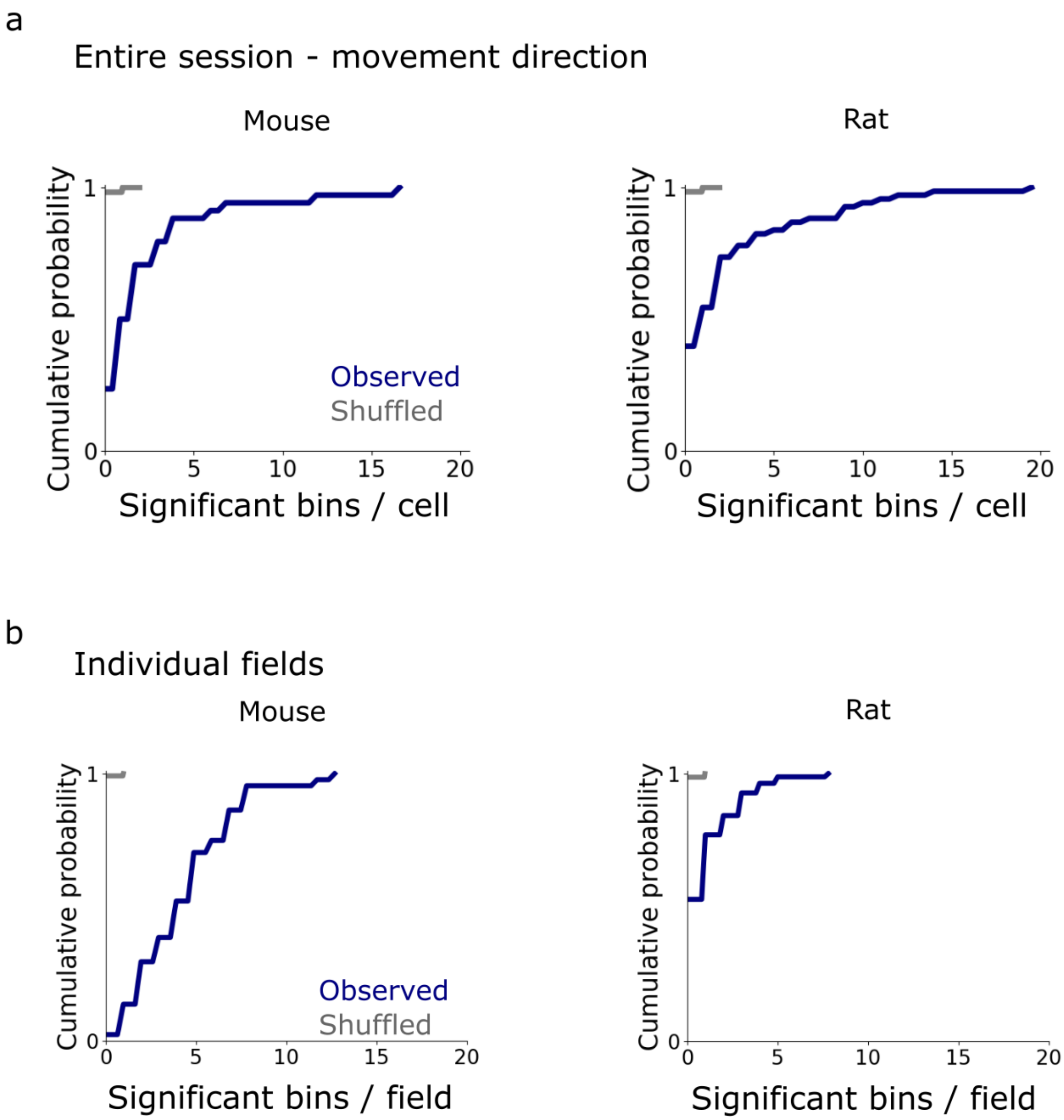

Supplementary Figure 3. Relationship between grid cell firing and movement direction. We repeated analyses from Fig. 2 and Fig. 3 but binning spikes as a function of movement direction [56] instead of head direction. **(a)** For analysis of global firing fields most grid cells were modulated by movement direction (26 / 34 grid cells for mice, and 41 / 68 for rats)(mice: 2.5 ± 3.5 bins / cell, rats: 2.6 ± 3.9 bins / cell) but the proportion is lower than when considering head direction (34 / 34 cells for mice and 56 / 68 for rats)(mice: 7.3 ± 3.9 bins / cell, rats: 4.1 ± 4.5 bins / cell). The observed number of significant bins per field differed significantly from the shuffled data (two-sided Mann-Whitney U test comparing the number of significant bins, p =2.4 x10^{-14} for mice and p = 2.3 x 10^{-17} for rats.) **(b)** Most individual fields are modulated by movement direction (43 / 44 grid fields in 13 grid cells modulated by movement direction in mice, and 39 / 83 fields in 25 cells in rats)(mice: 4.5 ± 2.9 bins / field; rats: 0.98 ± 1.3 bins / field). The proportions were similar to head direction for mice and lower than head direction for rats (38 / 44 fields in 13 cells for mice and 47 / 83 in 25 cells for rats)(mice: 4.3 ± 3.2 bins / field;

rats: 2.1 ± 2.8 bins / field). The number of significant bins per field differed from the shuffled data (two-sided Mann-Whitney U test comparing the number of significant bins, $p = 5.5 \times 10^{-26}$, U = 10.5 for mice and $p = 5.9 \times 10^{-14}$, U = 7.5 for rats).

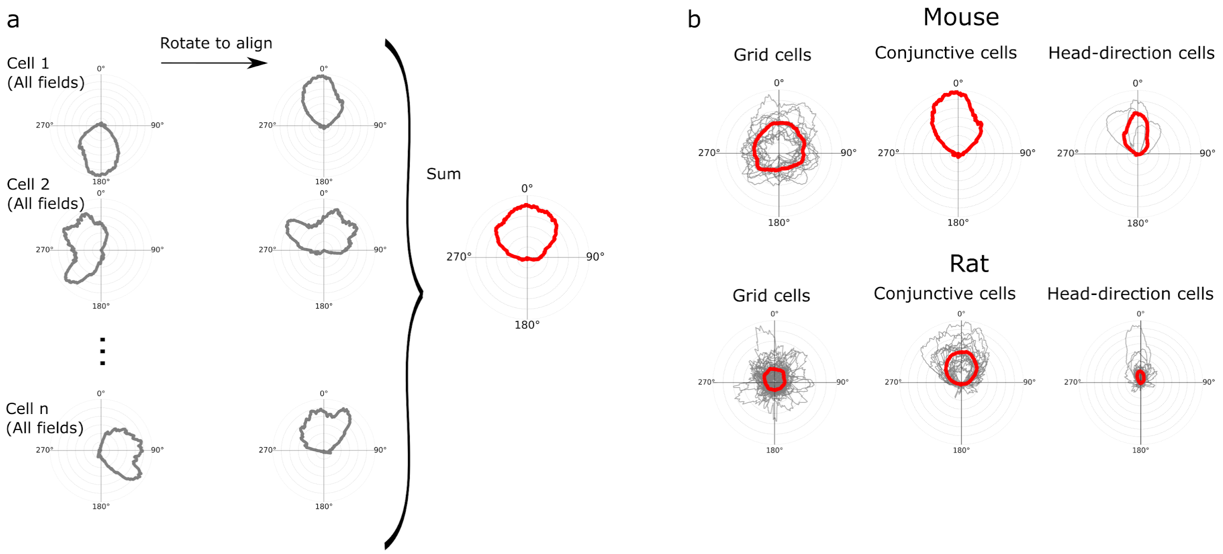

Supplementary Figure 4. Grid cell fields are not unidirectional. (a) Schematic of procedure for evaluating similarity of directional firing fields. Polar histograms of head direction dependent firing rates of data from all detected fields were rotated by the population mean vector of the combined distributions of head direction in all detected fields of a given cell to align them at 0. The examples shown here are for conjunctive cells. **(b)** Overlaid polar head direction histograms of grid cells, conjunctive cells and head direction cells with firing fields. Mean histograms are in red. In the histograms for grid cells the directionality appears smoothed by averaging indicating that there is no consistent shape to the firing field, whereas for head direction and conjunctive cells the averaged field appears unimodal.

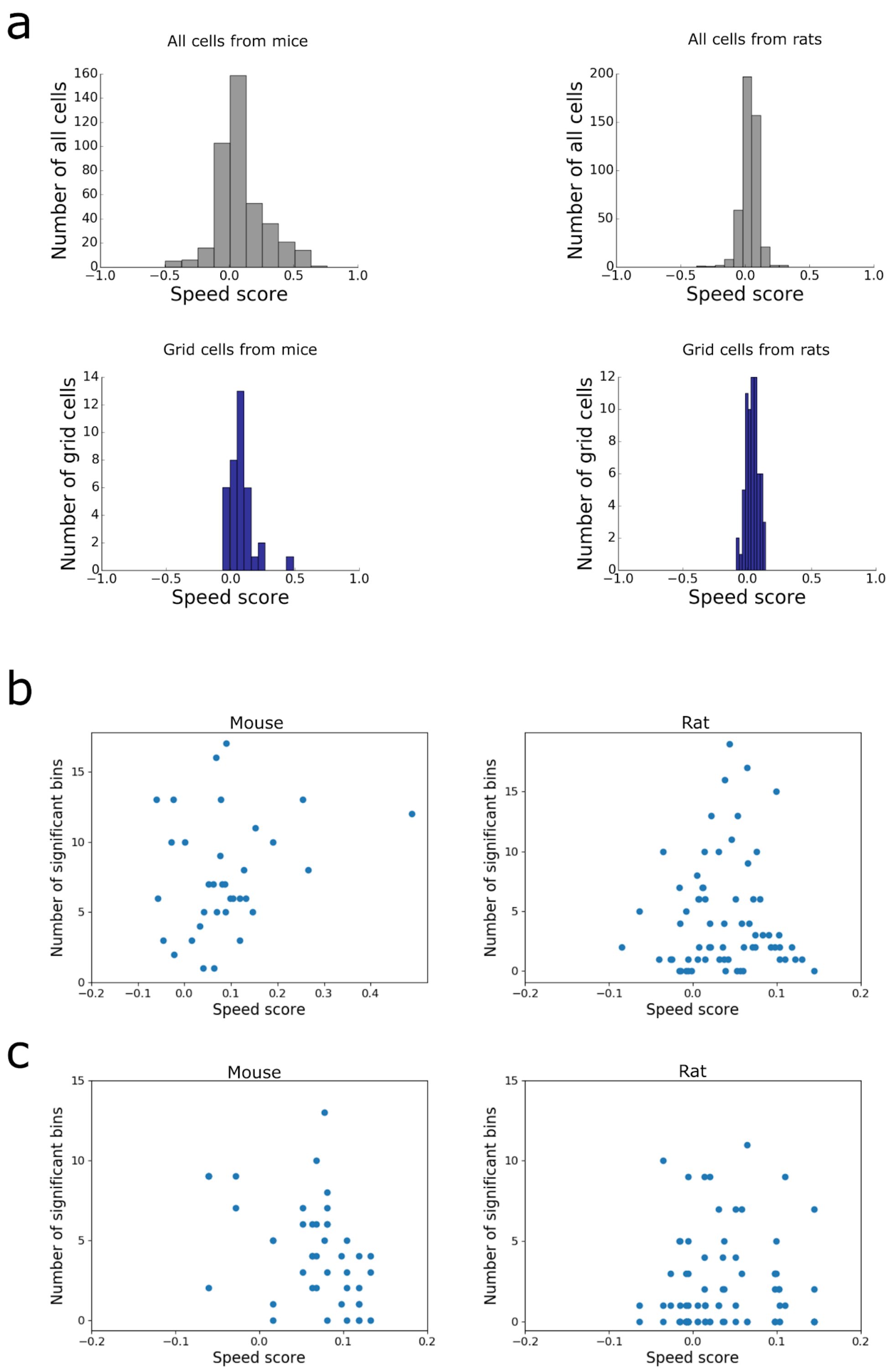

Supplementary Figure 5. Speed modulation is weak in most recorded grid cells. (a) Histograms of speed scores of all recorded cells (upper) and speed scores of grid cells only

(lower). **(b)** The number of significant directional bins / cell plotted as a function of speed score for grid cells from mice and rats. **(c)** The number of significant directional bins / field plotted as a function of speed score for grid cells from mice and rats. Note that data from (c) are from the subset of the grid cells in (b) from which fields could be automatically detected.

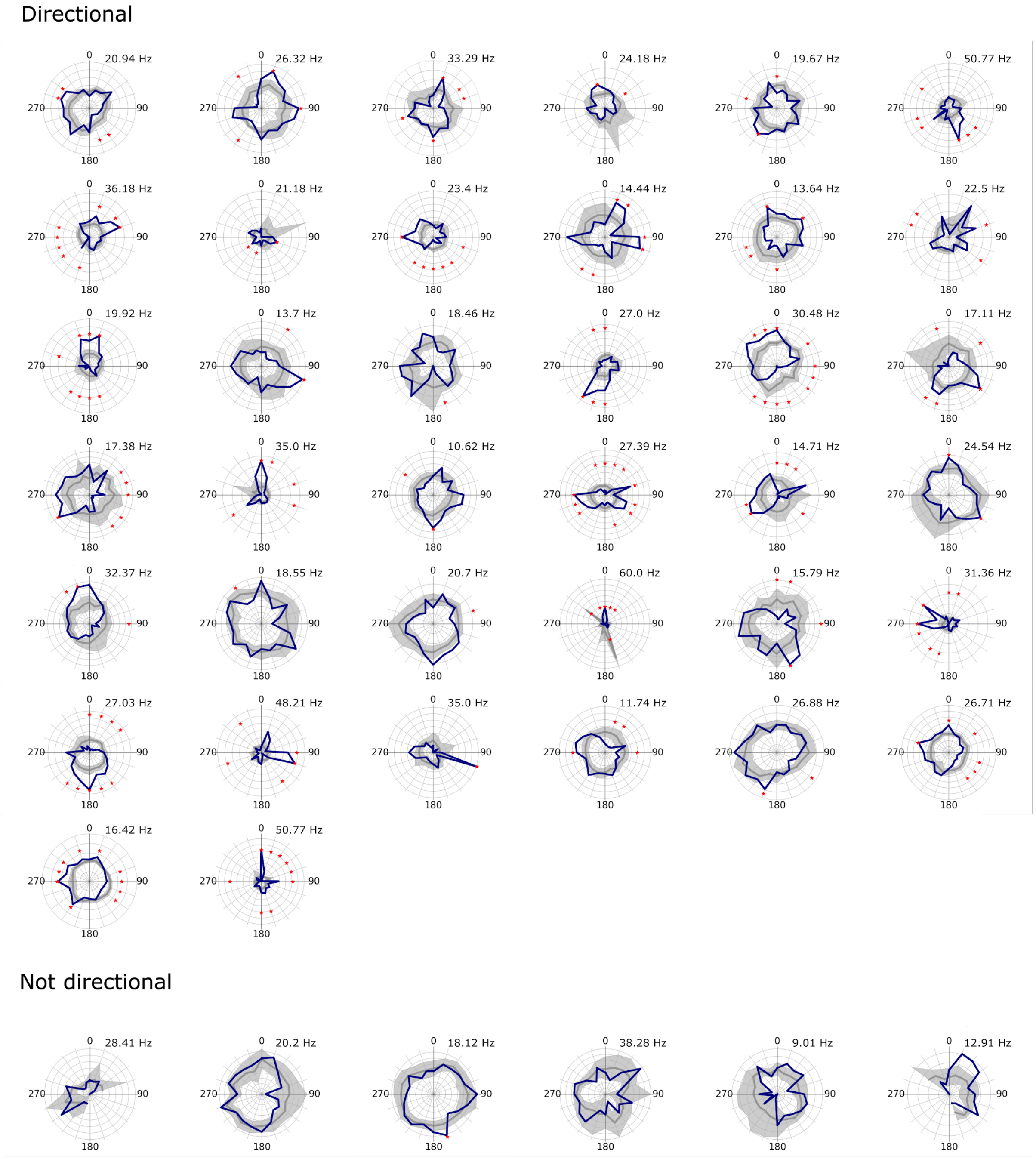

Supplementary Figure 6. Direction dependence of firing for individual fields from pure grid cells recorded from mice. The observed firing rate is shown binned as a function of head direction (blue) along with the corresponding shuffled data (grey) for each pure grid cell. The lighter grey region indicates the range of the 5th and 95th percentiles. The maximum firing rate of the observed data is shown above each plot. Significantly directional bars are marked with an asterix (*). Fields classified as 'directional' had at least one bin that differed statistically from the shuffled distribution ($p < 0.05$, two-tailed p value calculated from the shuffled distribution and

corrected for multiple comparisons with the Benjamini-Hochberg procedure), whereas field classified as ‘not directional’ did not.

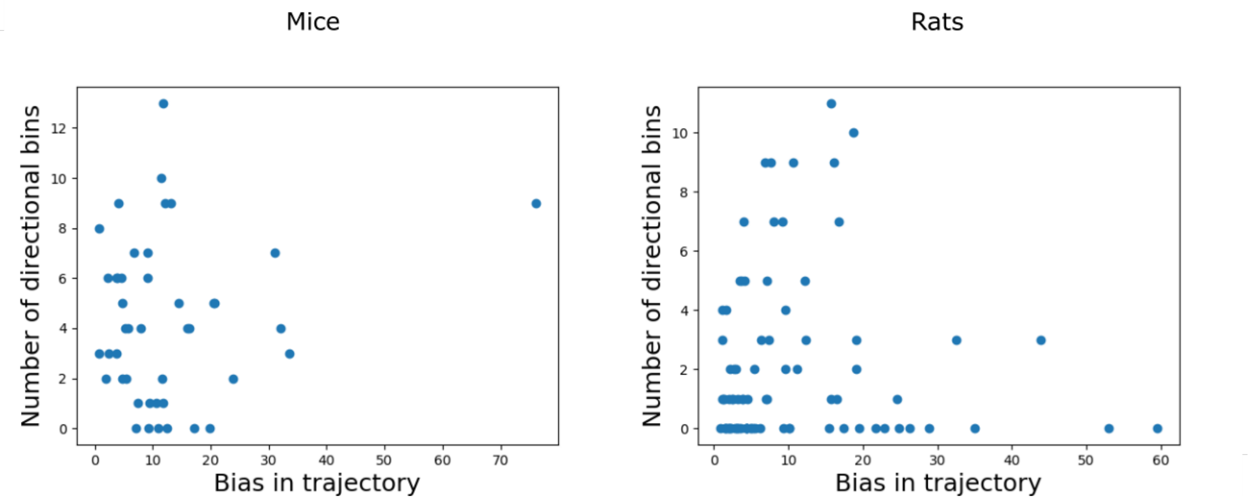

Supplementary Figure 7. The bias in the head direction of the trajectory does not correlate with directional tuning of firing by pure grid cells. The number of significant directional bins (out of 20 bins of the histogram) plotted against the one sample Watson test statistic (R, circular.watson_test) for each detected grid cell firing field. The trajectory bias did not correlate with head direction tuning (mice: slope = 0.035, two-sided $p > 0.37$, rats: slope = -0.00061, $p = 0.98$, Python,scipy.stats.linregress).

a

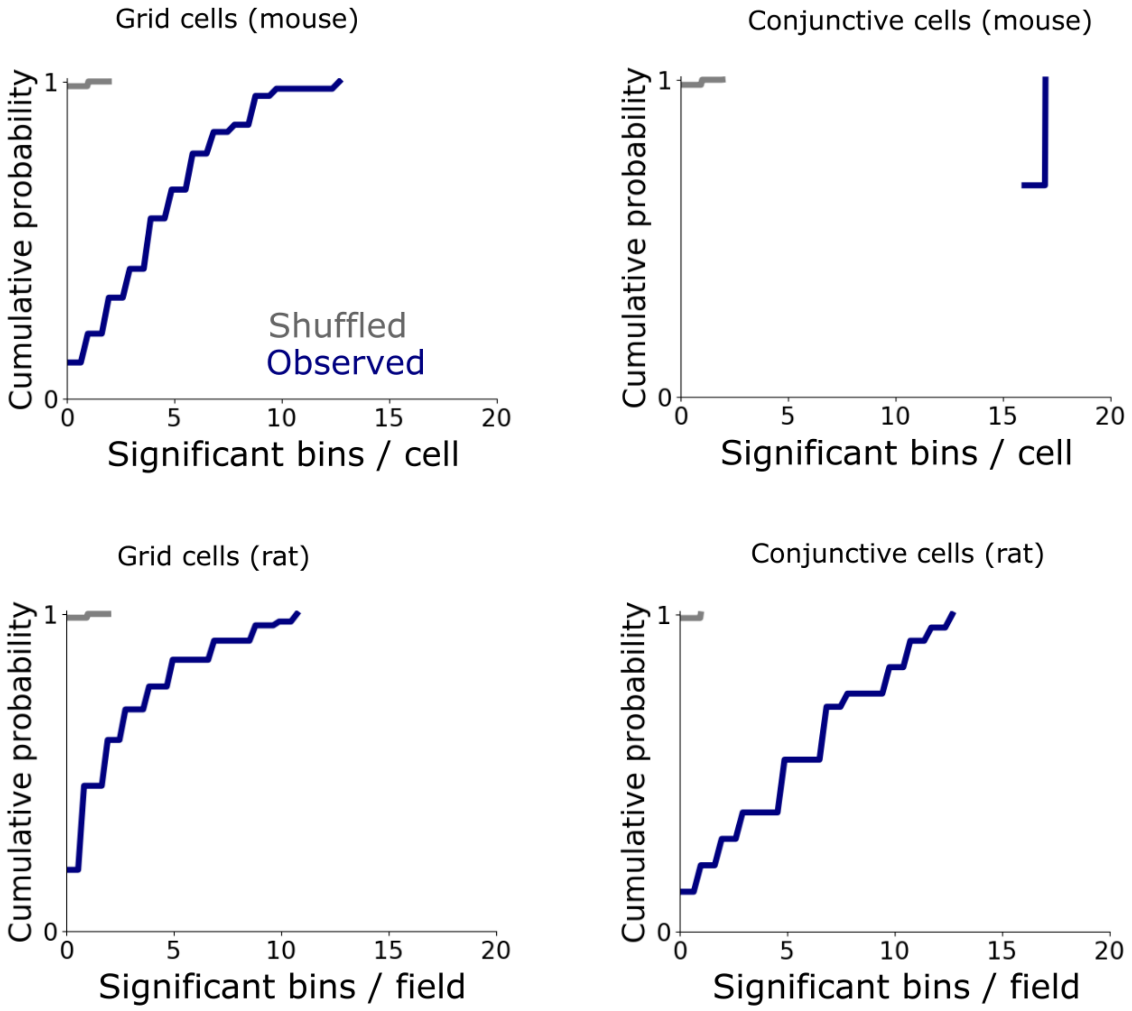

b

	Pure grid cells (mice)	Conjunctive cells (mice)	Pure grid cells (rats)	Conjunctive cells (rats)
Number of cells	13	1	25	24
Number of fields	44	3	83	7
Proportion of fields with significant bins	84.6 ± 27.1 %	100 % ± 0 %	61.7 ± 28.3 %	65.9 ± 31.8 %
Average number of significant bins	4.27 / 20 ± 3.15 in the observed data vs 0.15 / 20 ± 0.12 for the shuffled data	16.33 / 20 ± 0.47 in the observed data vs 0.012 ± 0.13 in the shuffled data	2.07 / 20 ± 2.83 in the observed data vs 0.012 / 20 ± 0.11 for the shuffled data	4.29 / 20 ± 4.09 in observed data in rats vs 0.012 / 20 ± 0.12 in shuffled data

Supplementary Figure 8. Comparison of directional firing in pure and conjunctive grid cells. (a) The directional dependence of spiking globally and at the level of single field was determined as in Fig. 2 and Fig. 3 respectively. The plots show the cumulative proportion of bins that differ significantly from their corresponding shuffled distribution globally (upper) and per field (lower) for pure grid cells (left) and conjunctive cells (right). (b) Summary data for proportion of fields with significant bins and average number of significant bins for pure and conjunctive grid cells.

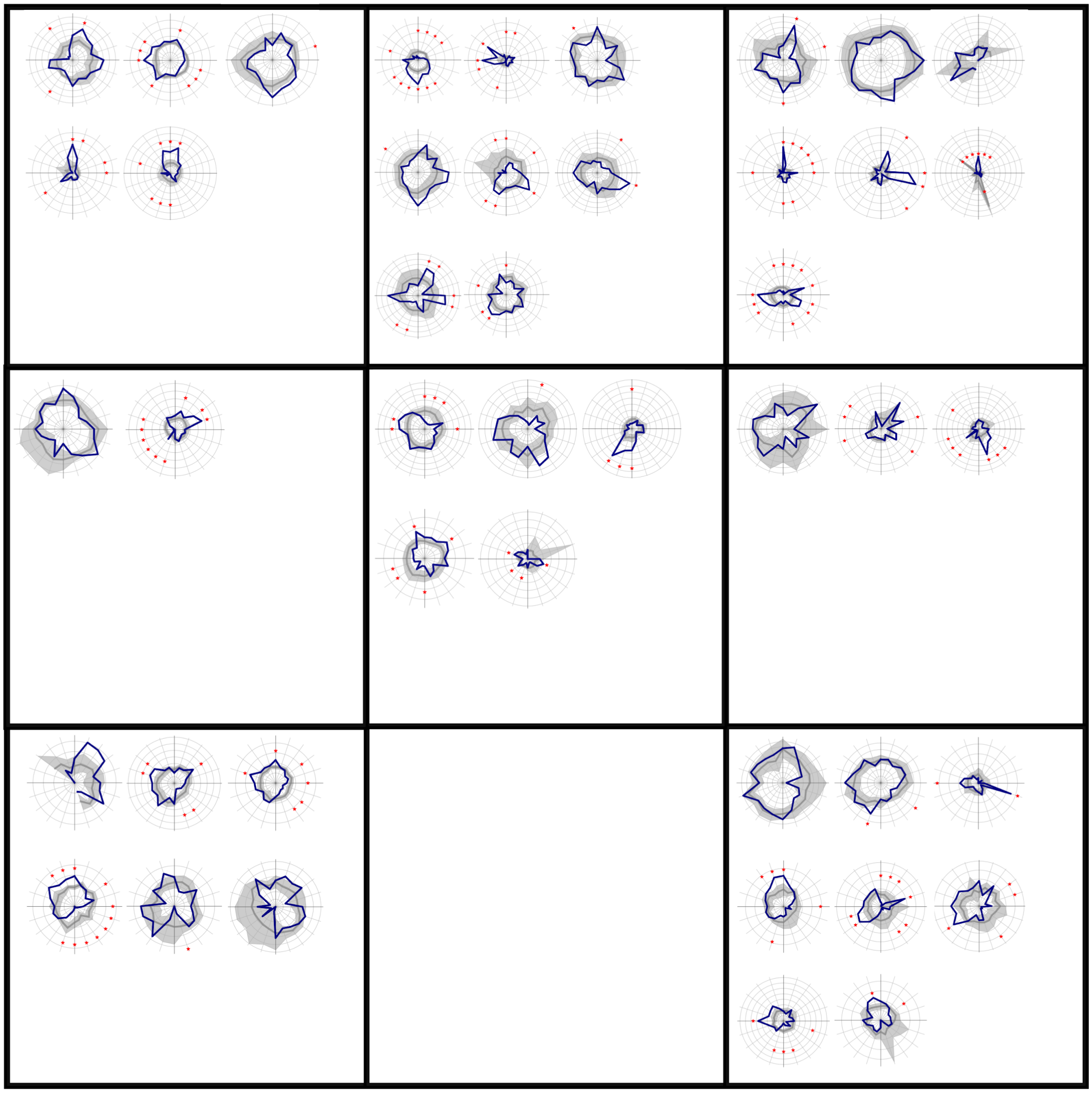

Supplementary Figure 9. Relationship between directional tuning and location in the arena. Distributive plots for all isolated mouse grid fields organised according to the location of the centre of each field in the recording arena.

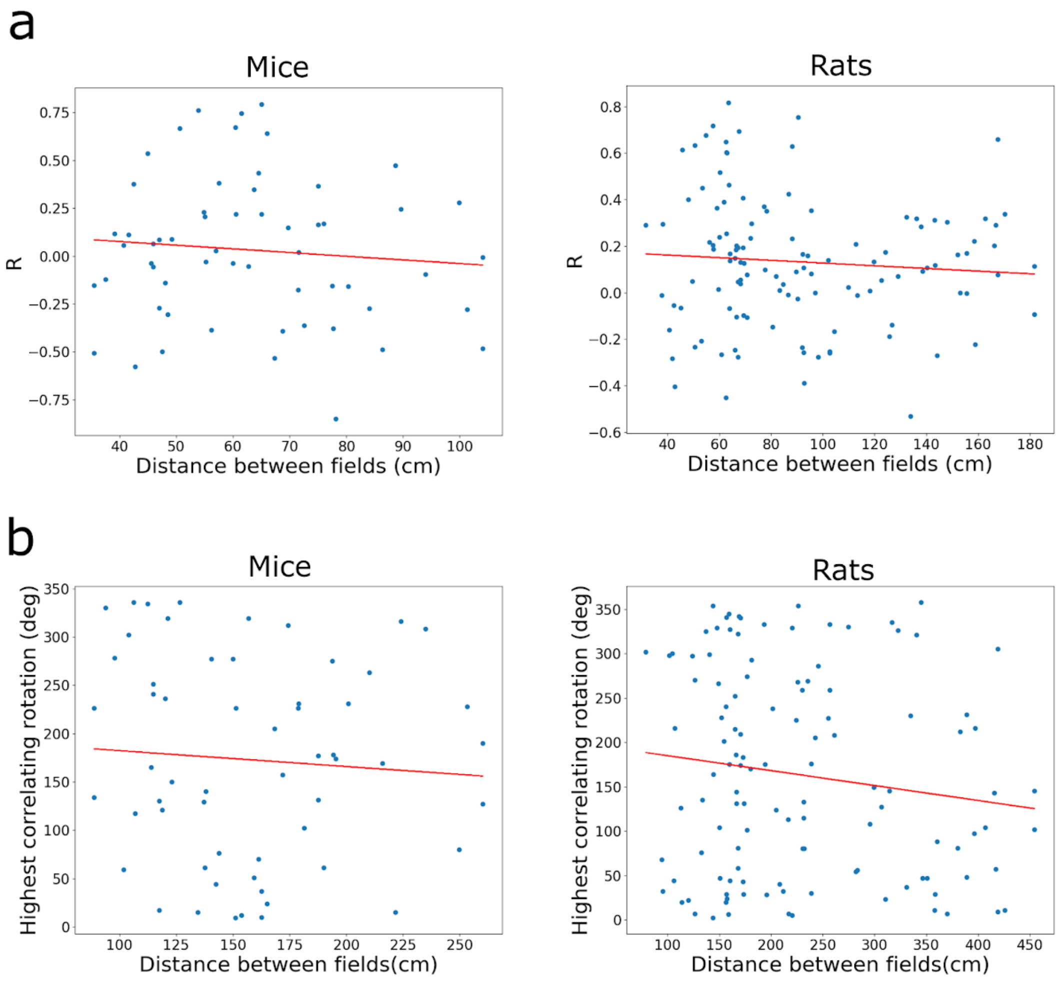

Supplementary Figure 10. Relationships between the separation of fields of pure grid cells and their directional preferences. (a) The Pearson correlation between classic head direction plots from pairs of grid fields, each from the same cell, plotted as a function of their distance from one another (mice: Pearson coefficient = -0.093, p = 0.32; rats: Pearson coefficient = -0.080, p = 0.22)(Linear regression results for mice: R = -0.093, p = 0.49, rats: R = -0.080, p = 0.39). (b) The angle of rotation resulting in the highest correlation of the directional histograms between pairs of grid fields plotted as a function of their distance from one another (mice: Pearson coefficient = 0.17, p = 0.45; rats: Pearson coefficient = 0.18, p = 0.13)(Linear regression results for mice: R = -0.072, p = 0.59, rats: R = -0.14, p = 0.13).

Oscillatory interference: Giocomo et al 2007

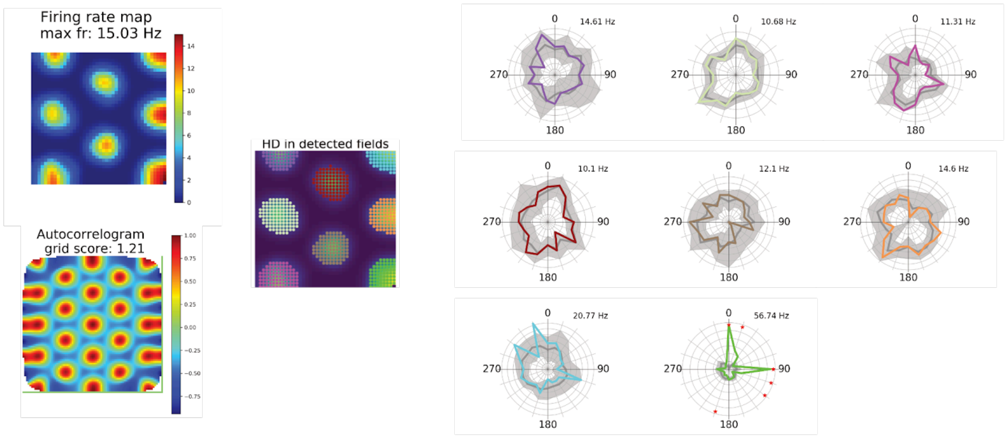

Continuous attractor: Guanella et al 2007

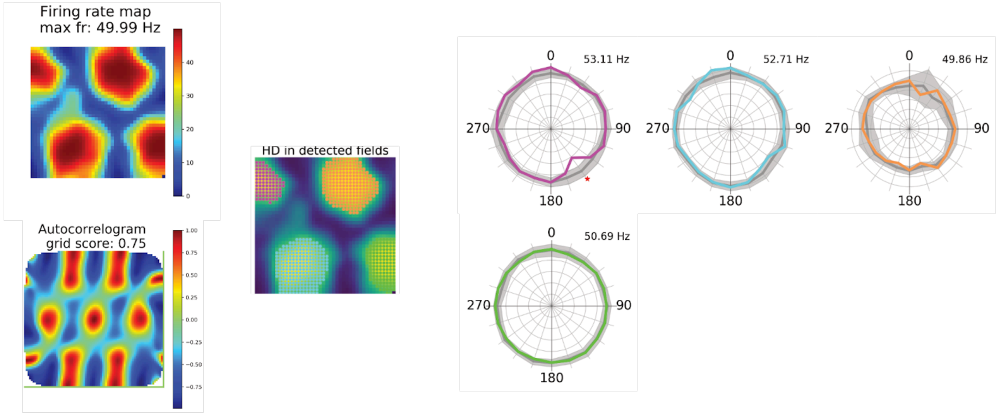

Supplementary Figure 11. Results from additional grid cell models. Firing rate map, autocorrelogram, and detected fields are shown on the left, plots of firing rate binned as a function of head direction shown on the right. Plots are generated as for Fig. 6.

a

	Experimental	Model
Resting membrane potential (mV)	-64.2±0.7	-63.12
Peak amplitude (mV)	41.9±1.7	42.19
Sag ratio	0.66±0.02	0.60
Half-width (ms)	0.59±0.02	0.55
Voltage threshold (mV)	-44.7±1.45	-57.35
Rheobase (pA)	181.8±18.8	181.6
Depolarization block (pA)	NA	92500
Input resistance (MΩ)	55.5±9.6	29.79

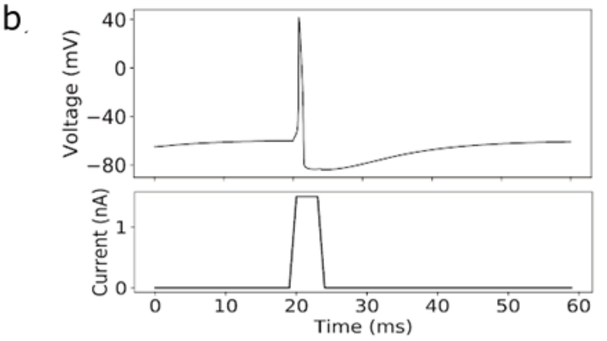

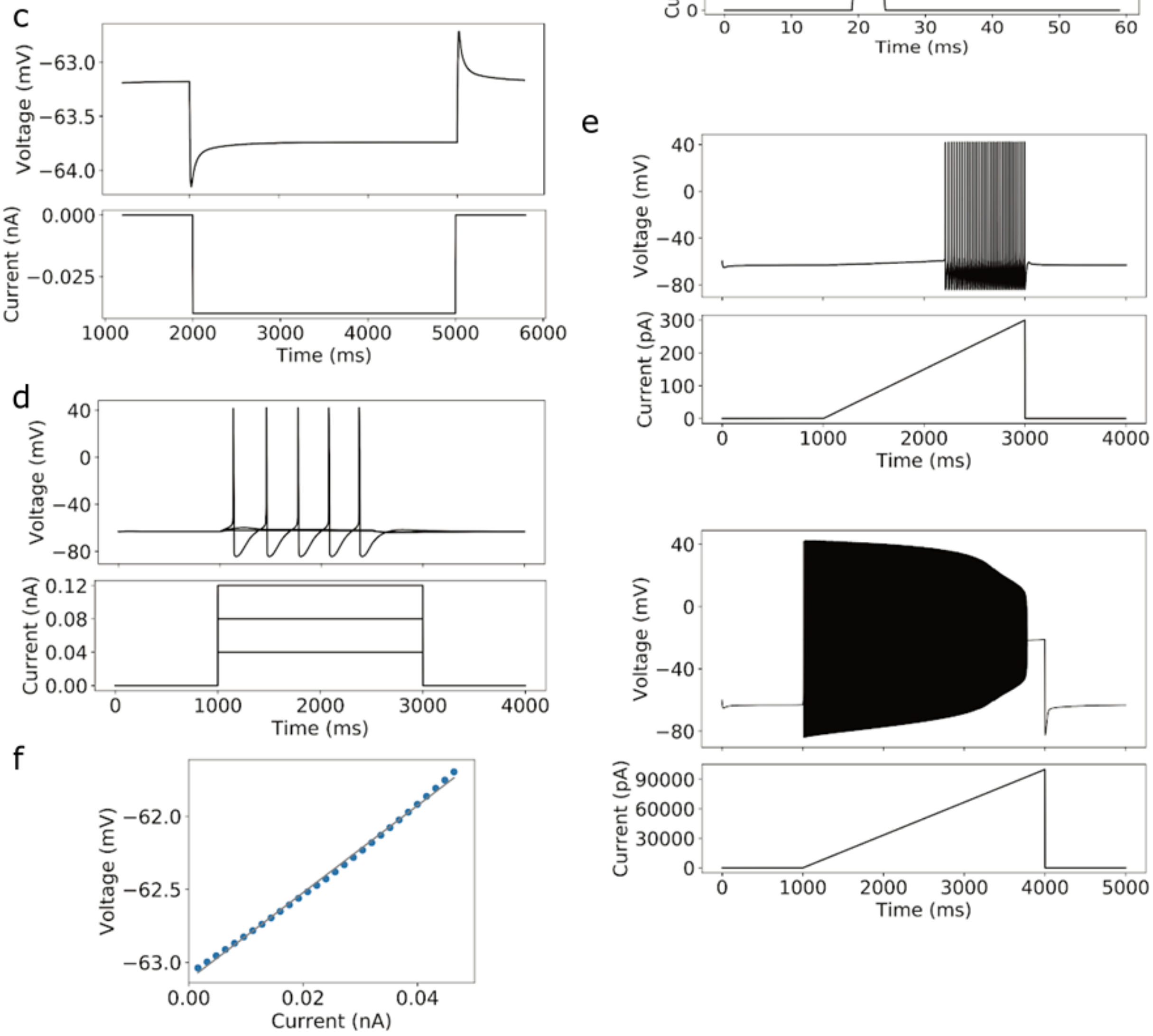

Supplementary Figure 12. Characterization of grid cell model. (a) Summary of electrophysiological properties of the model compared to experimental data [33]. **(b)** Single action potential used to determine peak-amplitude and half-width (upper) and injected current (lower). **(c)** Voltage response to injection of a 40 pA hyperpolarizing current used to determine the sag ratio. **(d)** Voltage responses (upper) to increasing current steps (lower) used to determine voltage threshold. **(e)** Voltage responses (upper panels in each pair) to injection of ramp

currents (lower panels in each pair) to determine the rheobase and depolarization block. **(f)** Determination of input resistance as the slope of the linear fit of steady-state voltage change induced by small current injections.

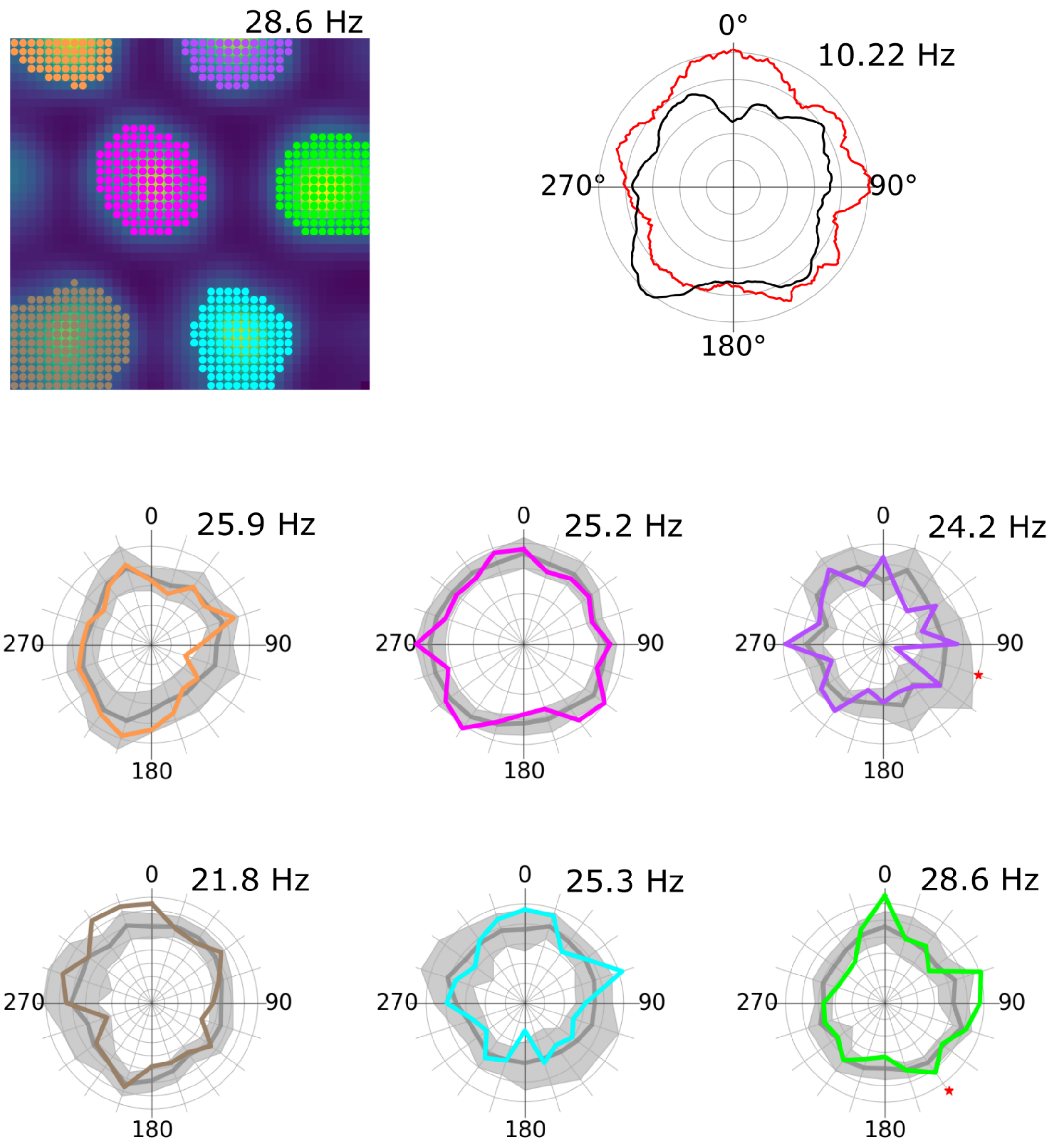

Supplementary Figure 13. Firing fields of simulated grid cells receiving input from conjunctive cells with spatially uniform firing rates. Example firing rate map (upper left), global head direction plot (upper right) and directional firing for individual fields (lower) for a model neuron receiving input from 5 aligned and conjunctive cells with spatially uniform firing rates.

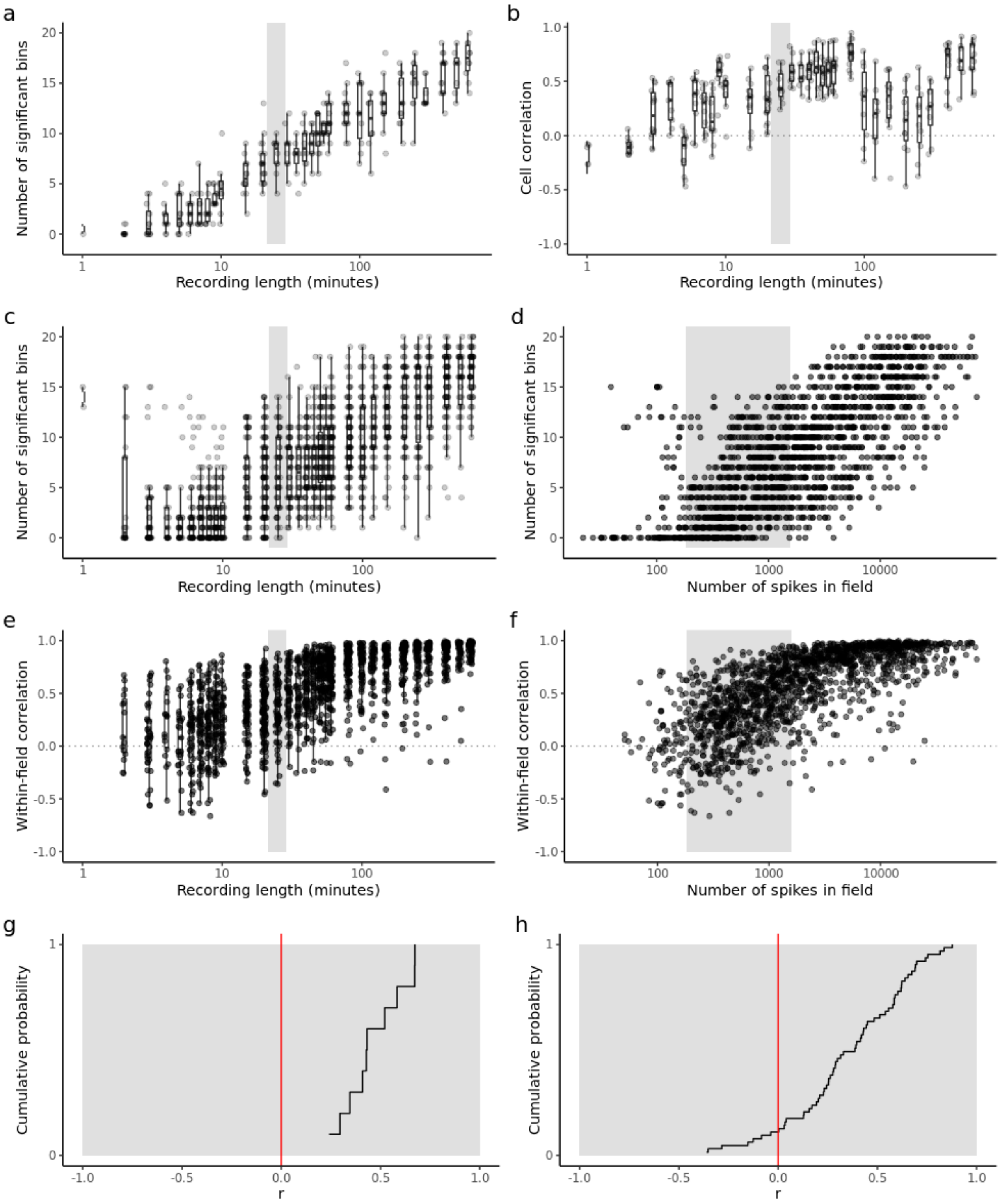

Supplementary Figure 14. Detection of directional firing and stability depend upon recording duration. We simulated the non-uniform conjunctive cell model with recording durations between 1 minute and 600 minutes (*n* = 10.8 ± 2.6 cells and *n* = 57.4 ± 20.9 fields per recording duration). Cells from the simulations were analysed to detect significant bins and correlations, followed by field-level analysis for significant bins and within-field correlations.

Long trajectories were created by concatenating experimental trajectories. The shaded region in each plot indicates the range of recording durations or number of spikes from the mouse experimental dataset. The mean/median ± sd values provided below are for simulations within that range. **(a)** Number of significant bins per cell as a function of recording duration (mean = 8.4 ± 5.3) . **(b)** Correlation of each cell's classic head direction plots from the first and second half of each session (median = 0.43 ± 0.15). **(c)** Number of significant bins per field as a function of recording duration (mean = 6.4 ± 3.9). **(d)** Number of significant bins per field as a function of the number of spikes in a field (mean = 4.9 ± 3.9). **(e)** Correlation of each field's classic head direction plot from the first and second half of each session as a function of recording duration (median = 0.39 ± 0.29) . **(f)** As for (e) but as a function of the number of spikes (median = 0.37 ± 0.31 in recording range). **(g)** Cumulative probability of global Pearson correlation coefficients as calculated in (b) for simulated cells in the experimental recording range. **(h)** Cumulative probability of local Pearson correlation coefficients as calculated in (e) for simulated fields in the experimental recording range.

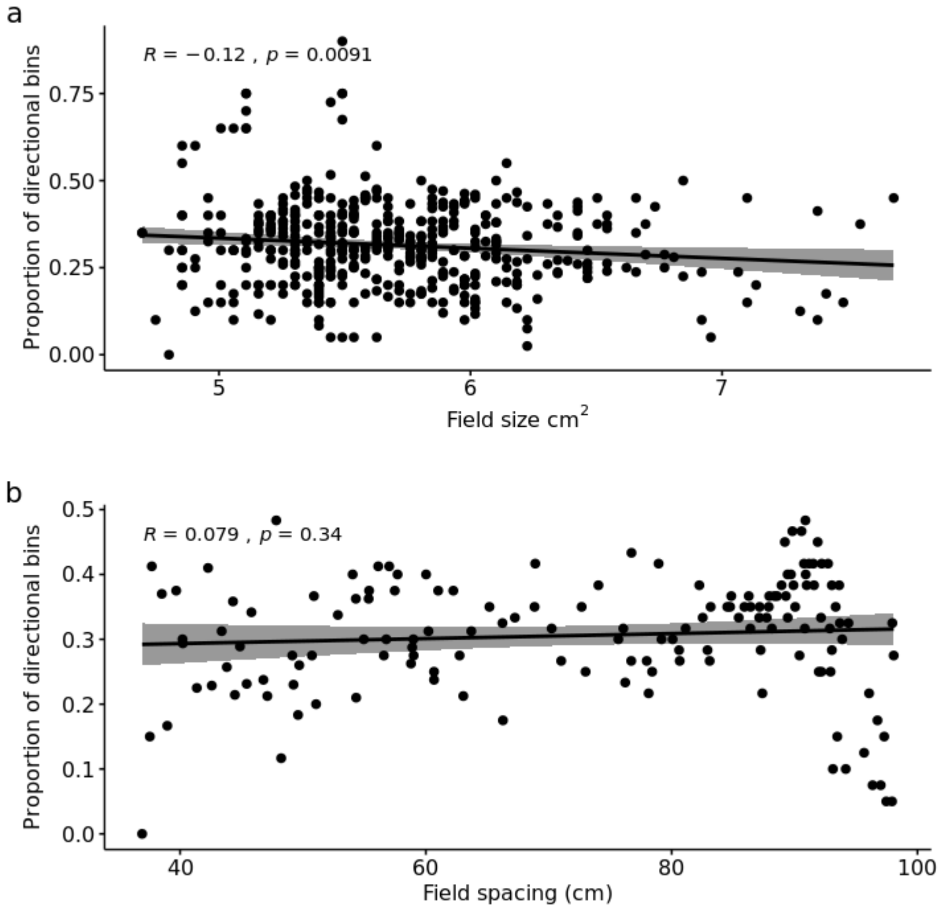

Supplementary Figure 15. **Relationship between field size and field spacing and the proportion of directional bins detected in non-uniform conjunctive input models.** Simulations of the non-uniform conjunctive cell input models were run with different field spacing or field sizes. Each point is a cell, because field spacing and field size are metrics calculated per-cell, rather than per field. (a) Field size had a weak but statistically significant effect on the proportion of significant bins detected (Pearson coefficient = -0.12, p = 0.0091). (b) Field spacing had no effect on the proportion of significant bins detected (Pearson coefficient = 0.079, p = 0.34). The shaded region indicates the 95 % confidence interval.

Supplementary Notes

Supplementary Note 1. Tetrode recording location for each experimental animal from in vivo open field experiments. 50 um sagittal sections were stained with NeuroTrace and expression of AAV-TRE-ChR2-mCherry was enhanced with anti-mCherry. Sections were imaged at 10x objective by Zeiss Axio Scan Z1. The scale bars correspond to 500 μm. The scale bar on the first panel of each page also applied to all subsequent panels.

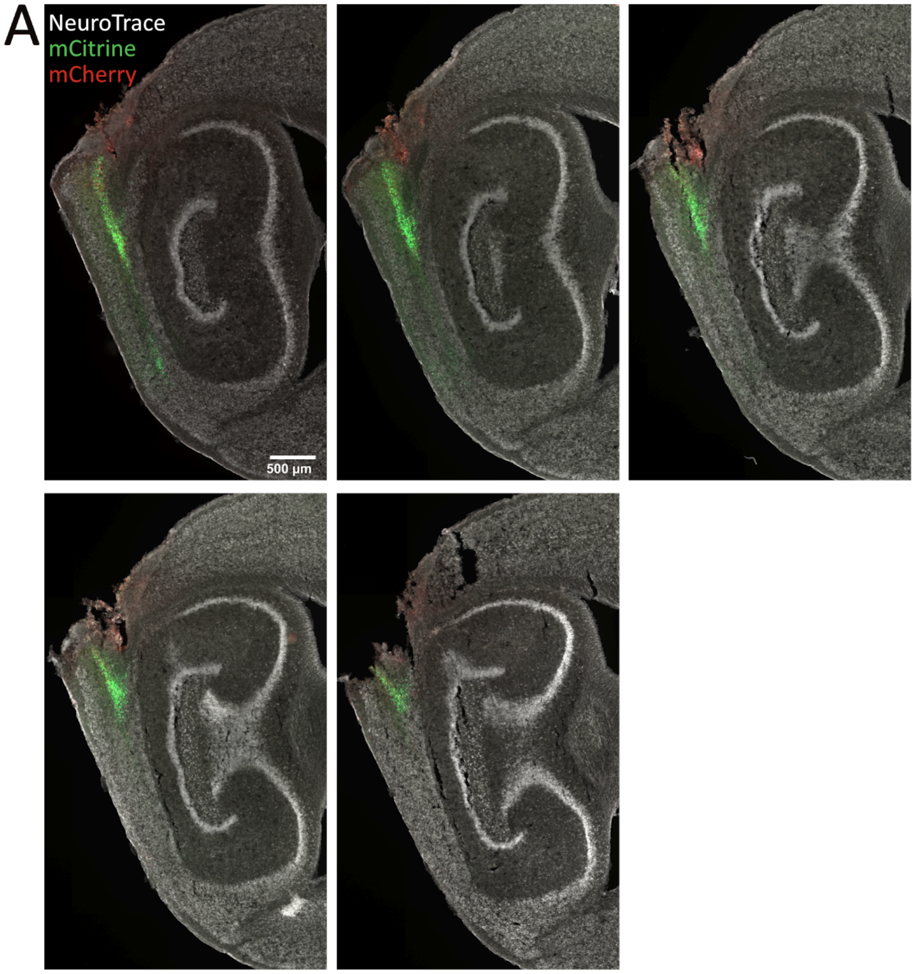

B

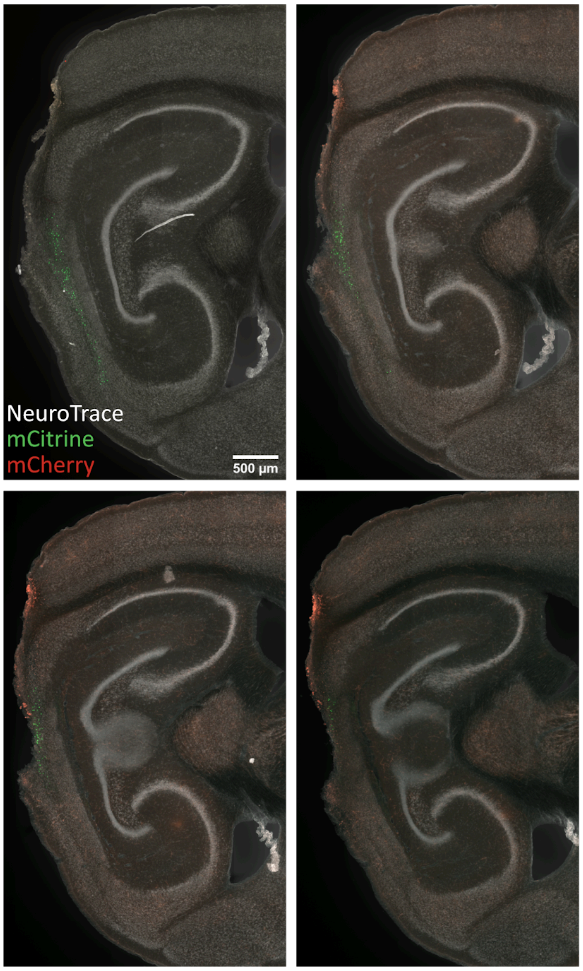

C

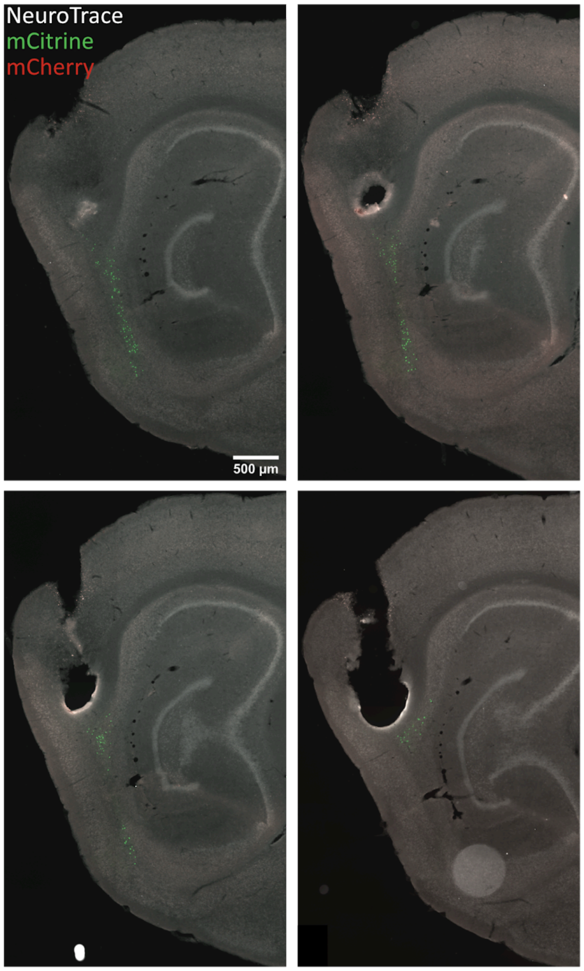

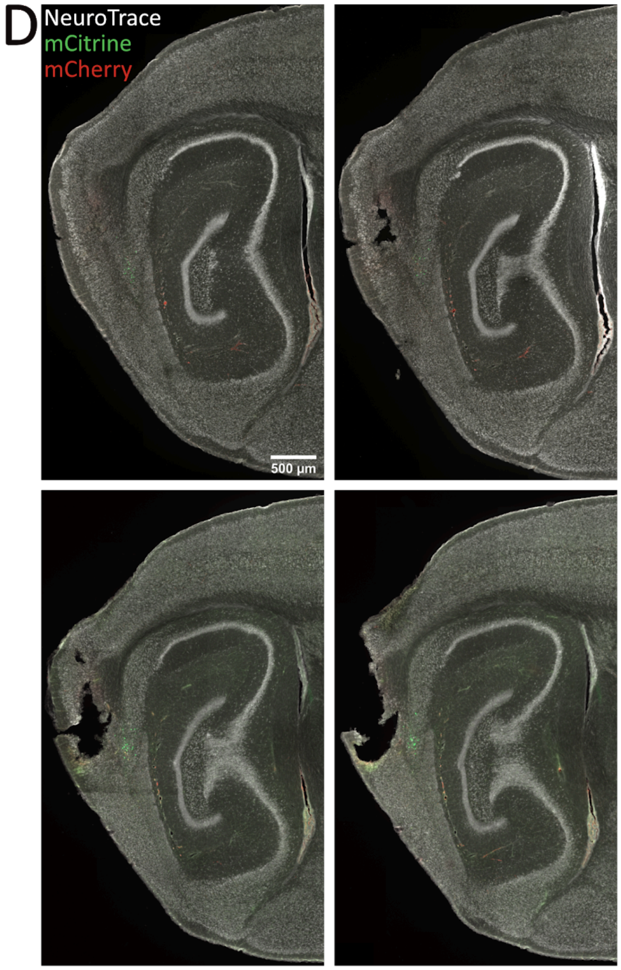
D
NeuroTrace
mCitrine
mCherry
500 μm

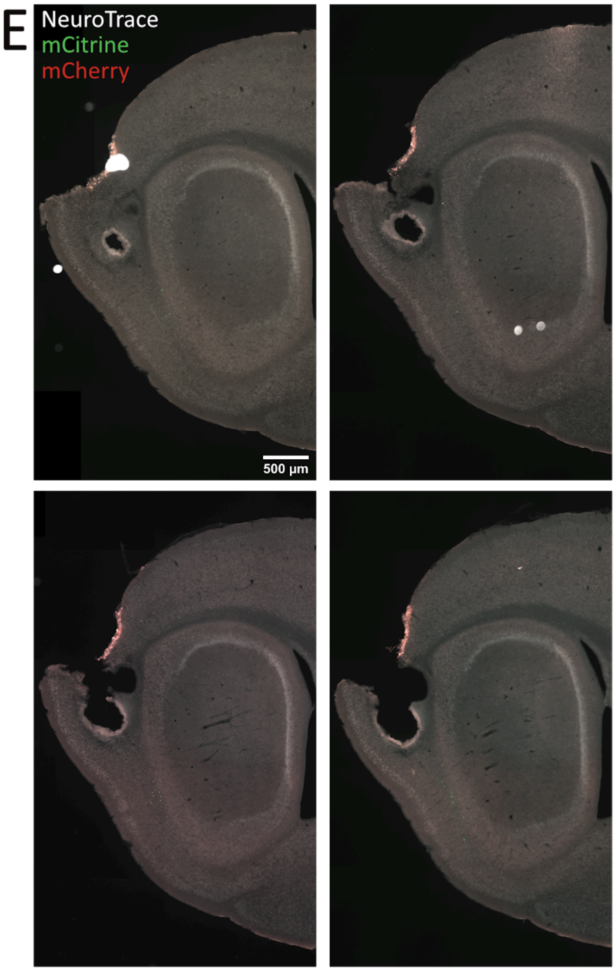
E
NeuroTrace
mCitrine
mCherry
500 µm

F

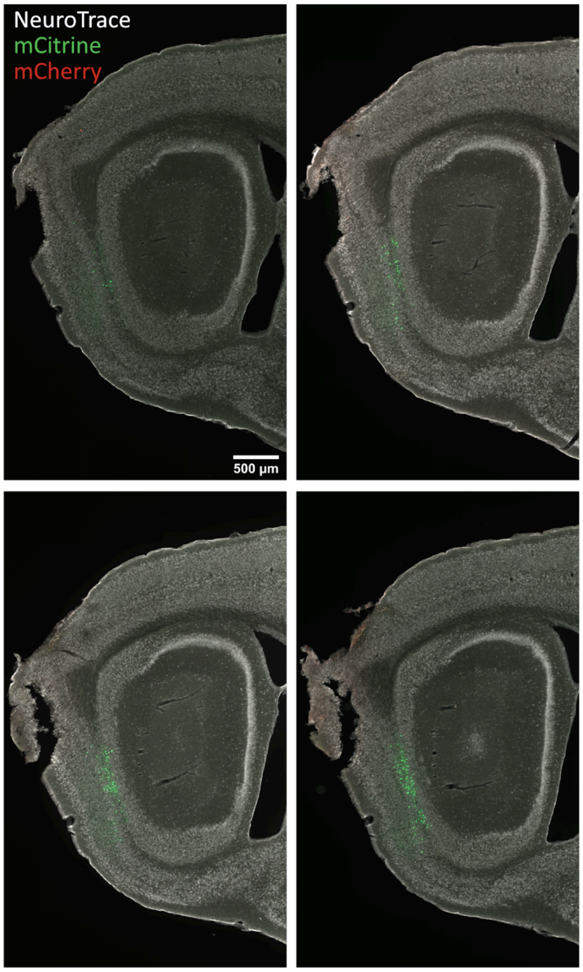

G

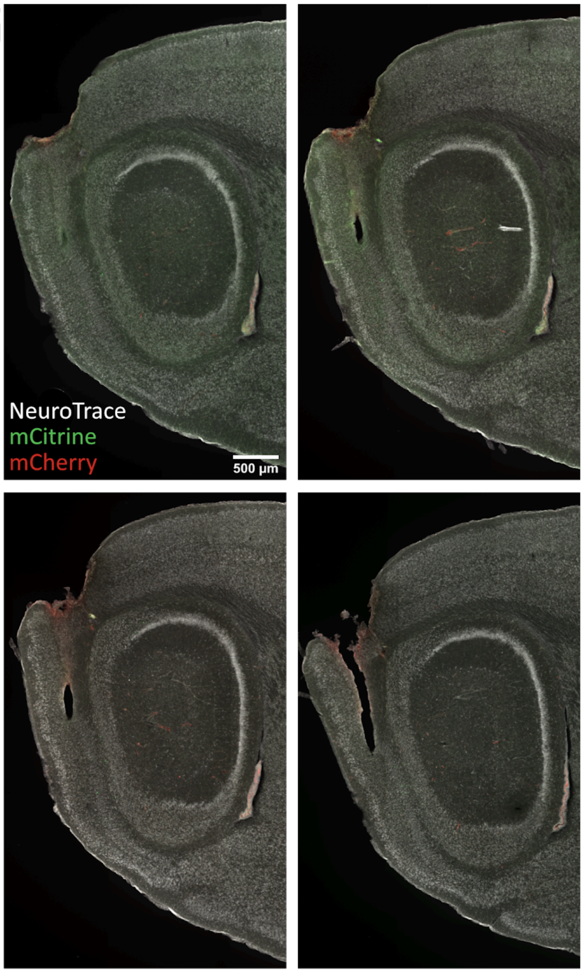

H

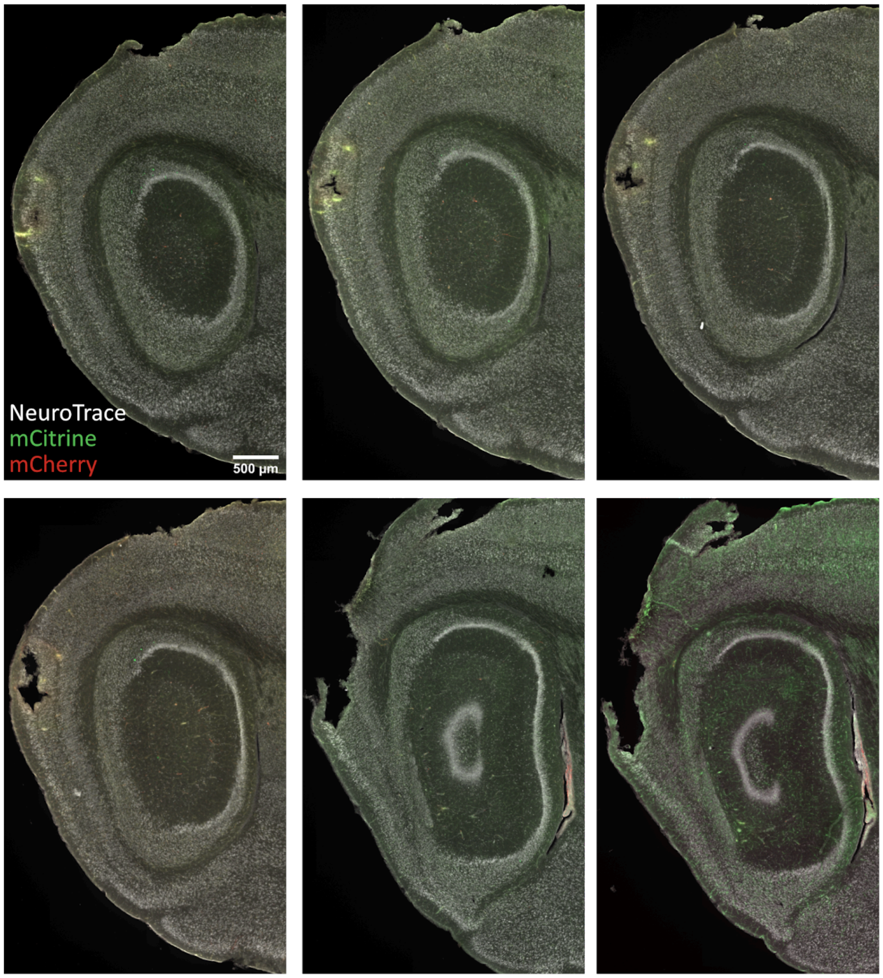

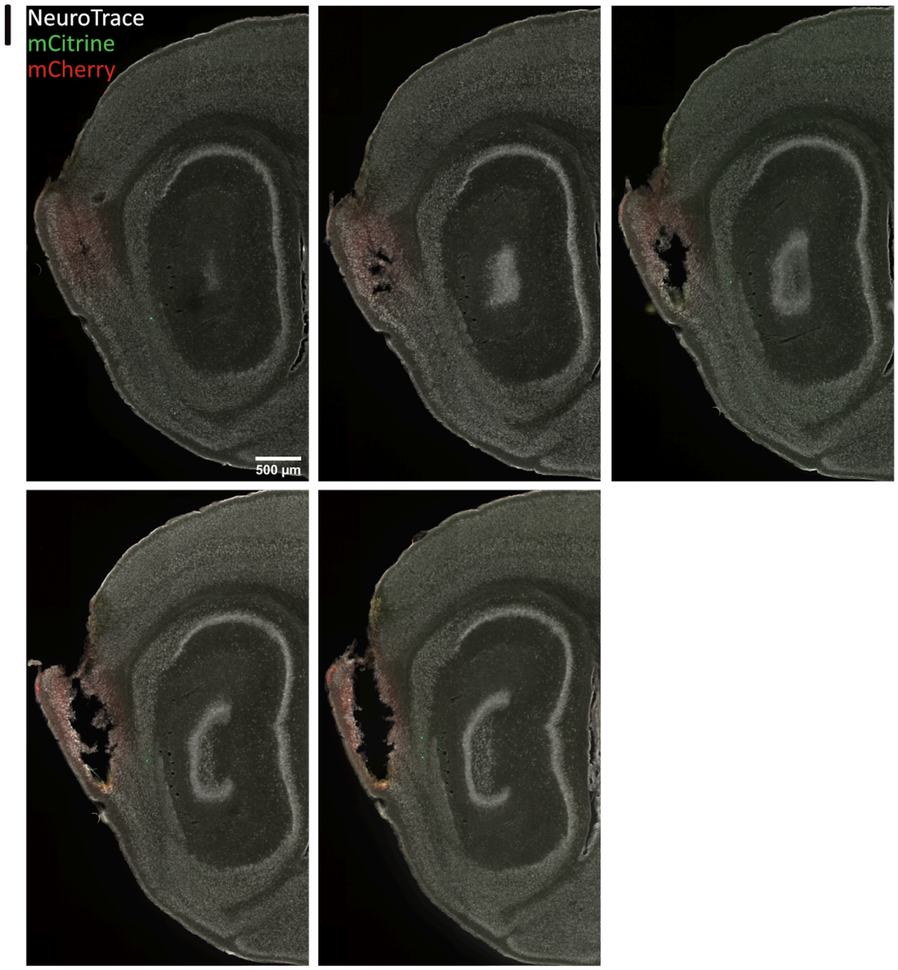
I
NeuroTrace
mCitrine
mCherry
500 µm

J

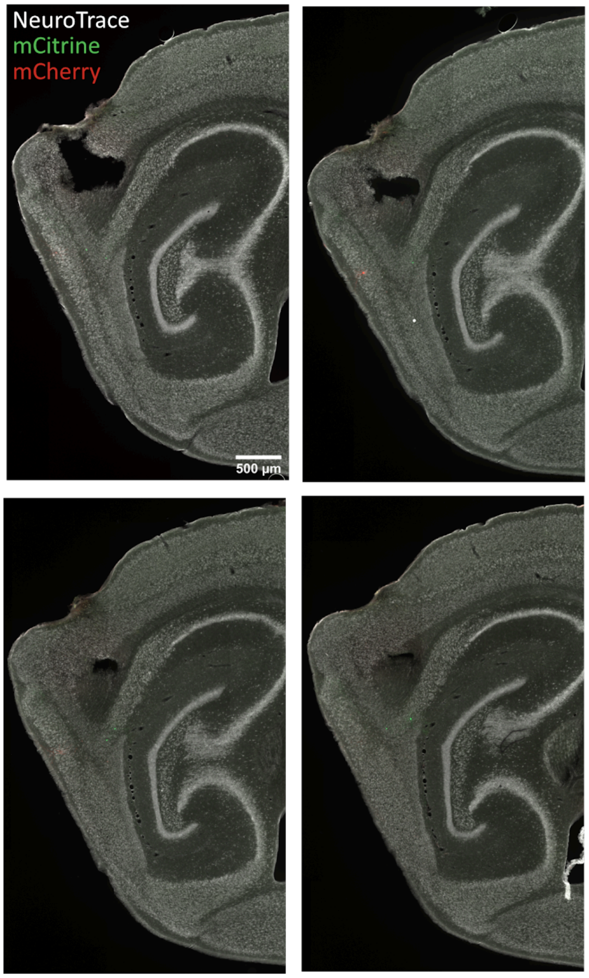

K

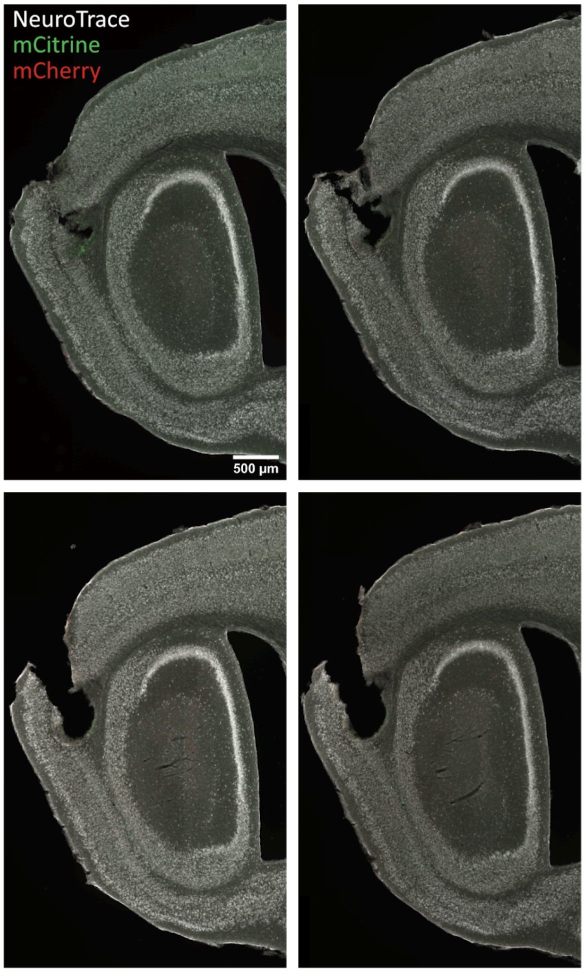

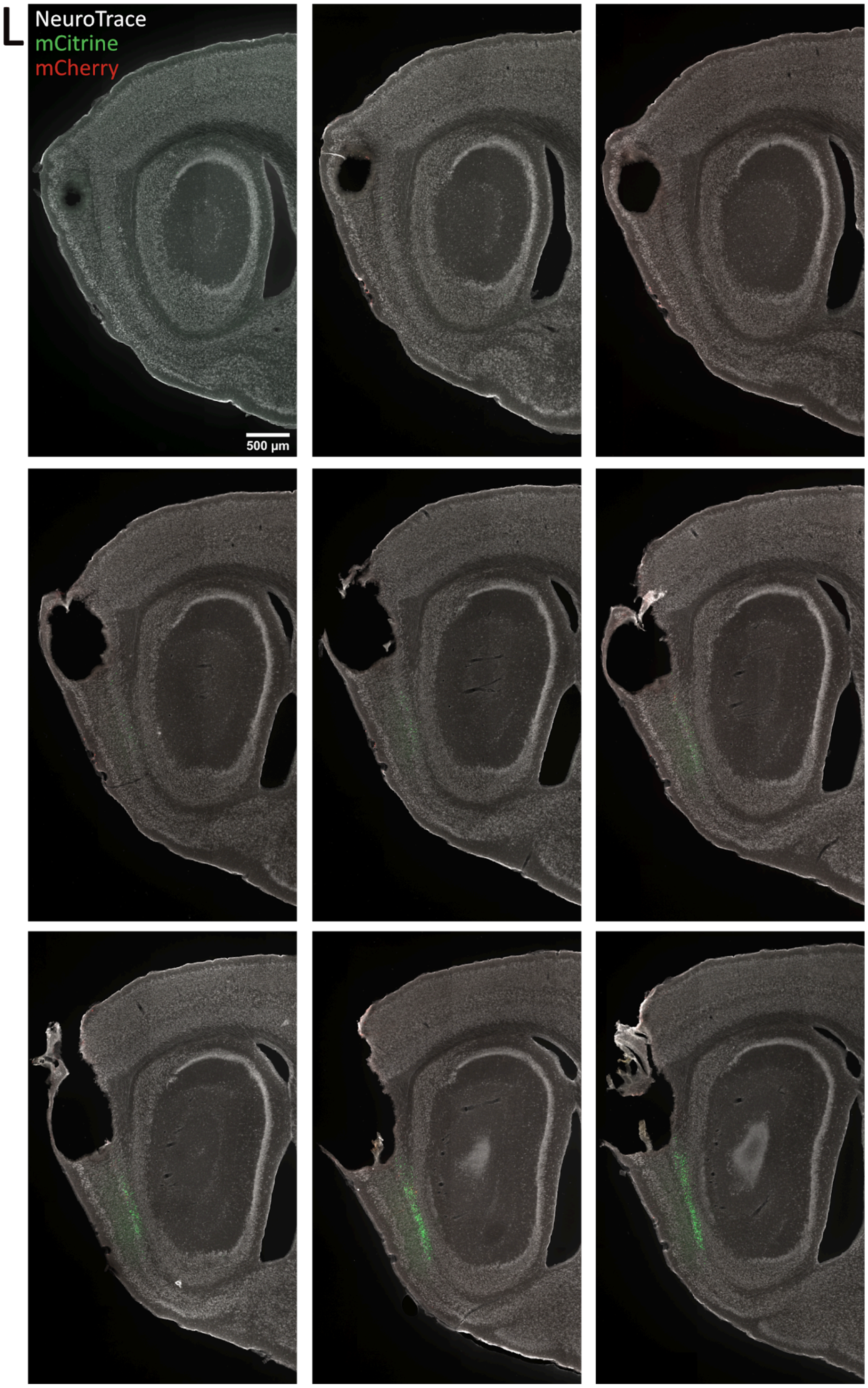
L
NeuroTrace
mCitrine
mCherry
500 µm

M

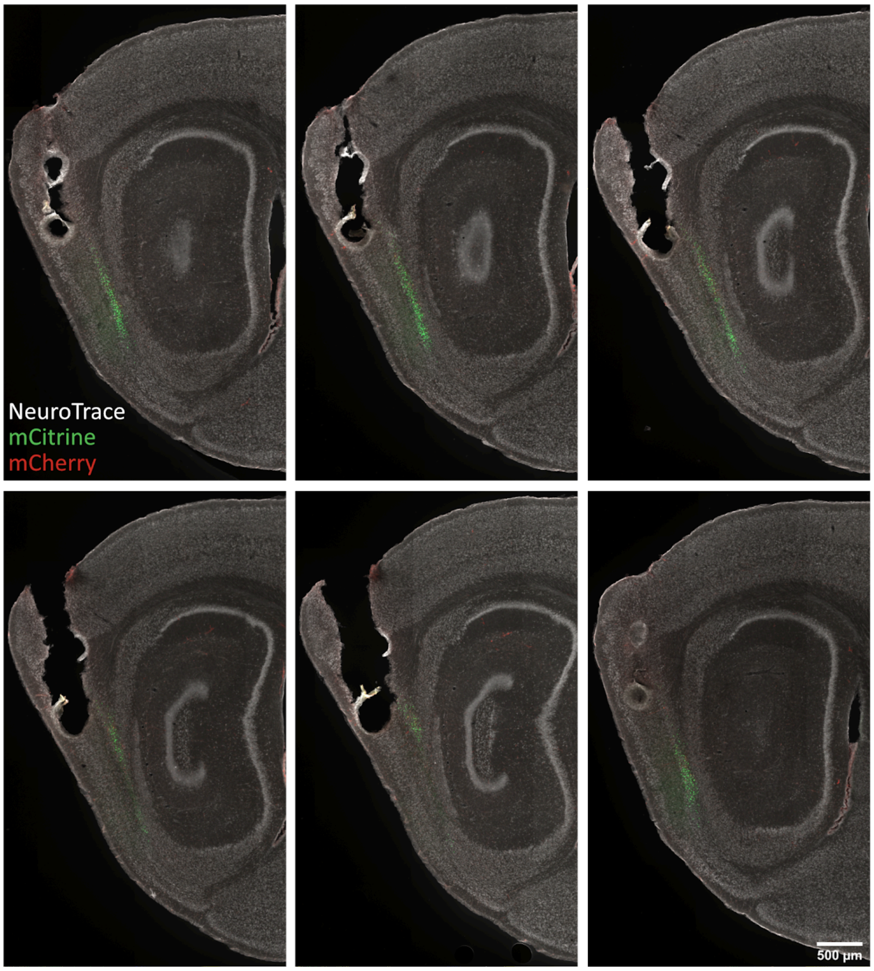

N

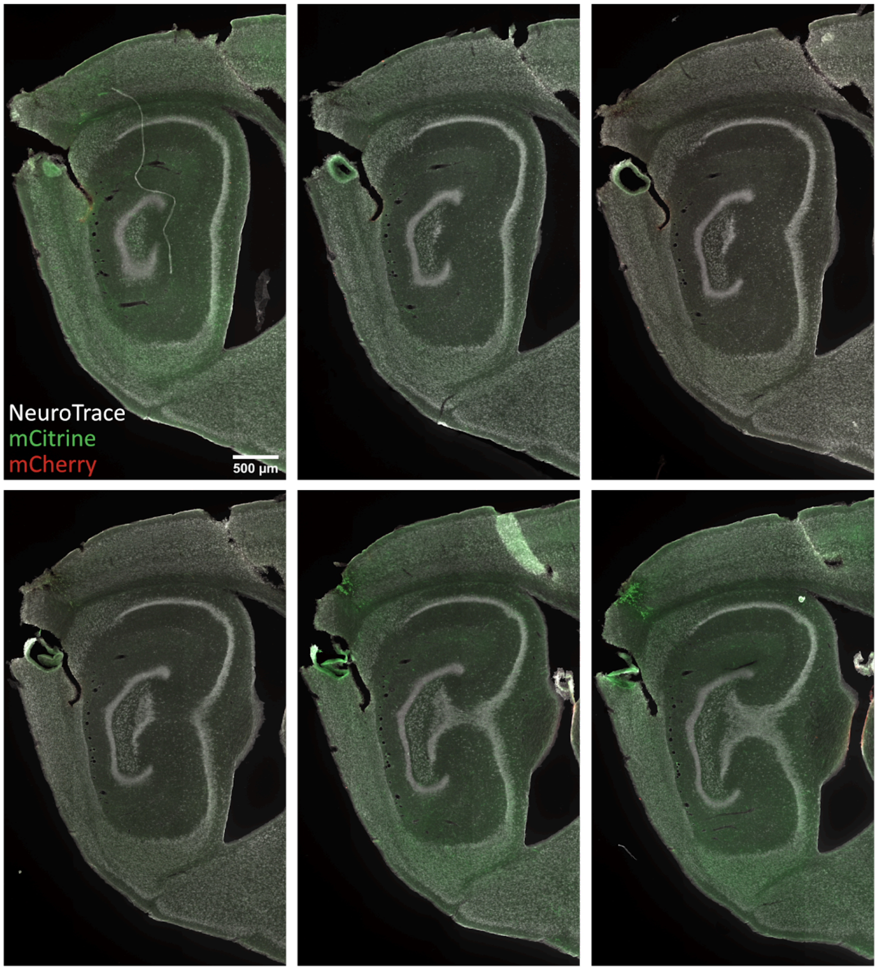

Supplementary Note 2. Mouse grid cells with more than two successfully detected firing fields. Each series of plots summarises data from a single grid cell. Top row left to right: action potential waveforms overlaid for the four channels of the tetrode, autocorrelograms of spike times, histogram of spike counts as a function of recording time, plot of spike rate as a function of movement speed, and coverage heat map for the position of the animal in the arena. Second row left to right: trajectory of the animal (black line) and firing events (red dots), firing rate map, autocorrelation matrix for the rate map, smoothed polar histogram of head direction when the cell fired (red, Hz) and from the whole session (black), scatter plot of firing events colour-coded for head direction on trajectory. Third row left to right: Detected firing fields on rate map and polar histograms of head direction in detected fields.

Grid cells
Animal ID: F

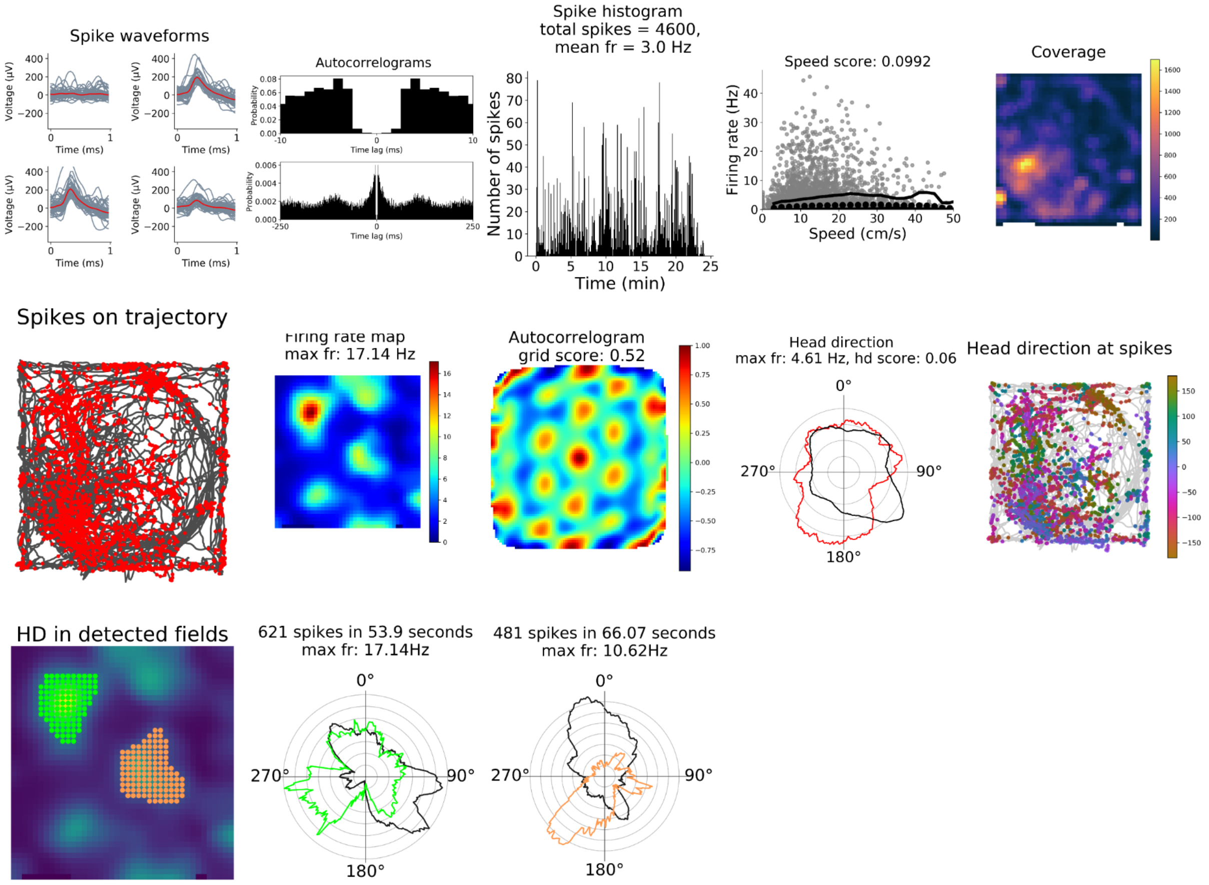

Animal ID: F

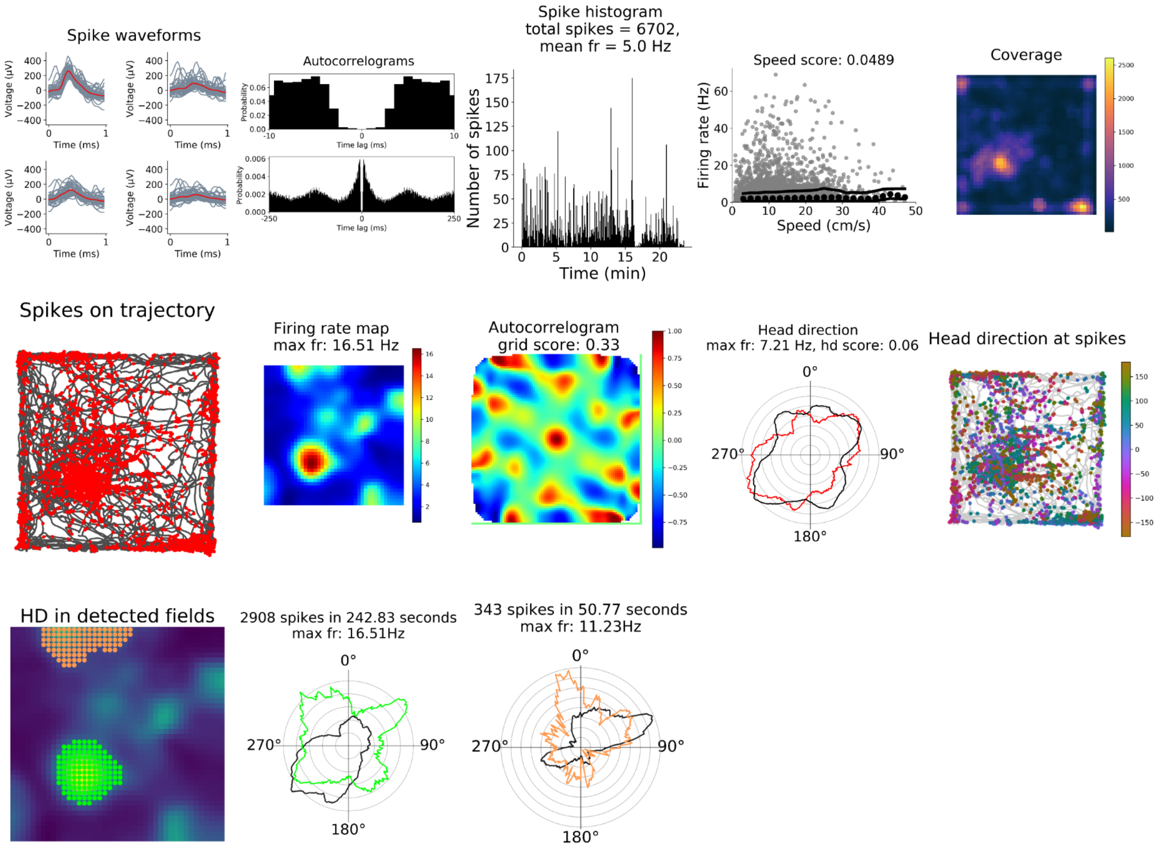
Spike waveforms
Voltage (µV)
Time (ms)
Autocorrelograms
Probability
Time lag (ms)
Spike histogram
total spikes = 6702,
mean fr = 5.0 Hz
Number of spikes
Time (min)
Speed score: 0.0489
Firing rate (Hz)
Speed (cm/s)
Coverage
Spikes on trajectory
Firing rate map
max fr: 16.51 Hz
Autocorrelogram
grid score: 0.33
Head direction
max fr: 7.21 Hz, hd score: 0.06
Head direction at spikes
HD in detected fields
2908 spikes in 242.83 seconds
max fr: 16.51Hz
343 spikes in 50.77 seconds
max fr: 11.23Hz

Animal ID: F

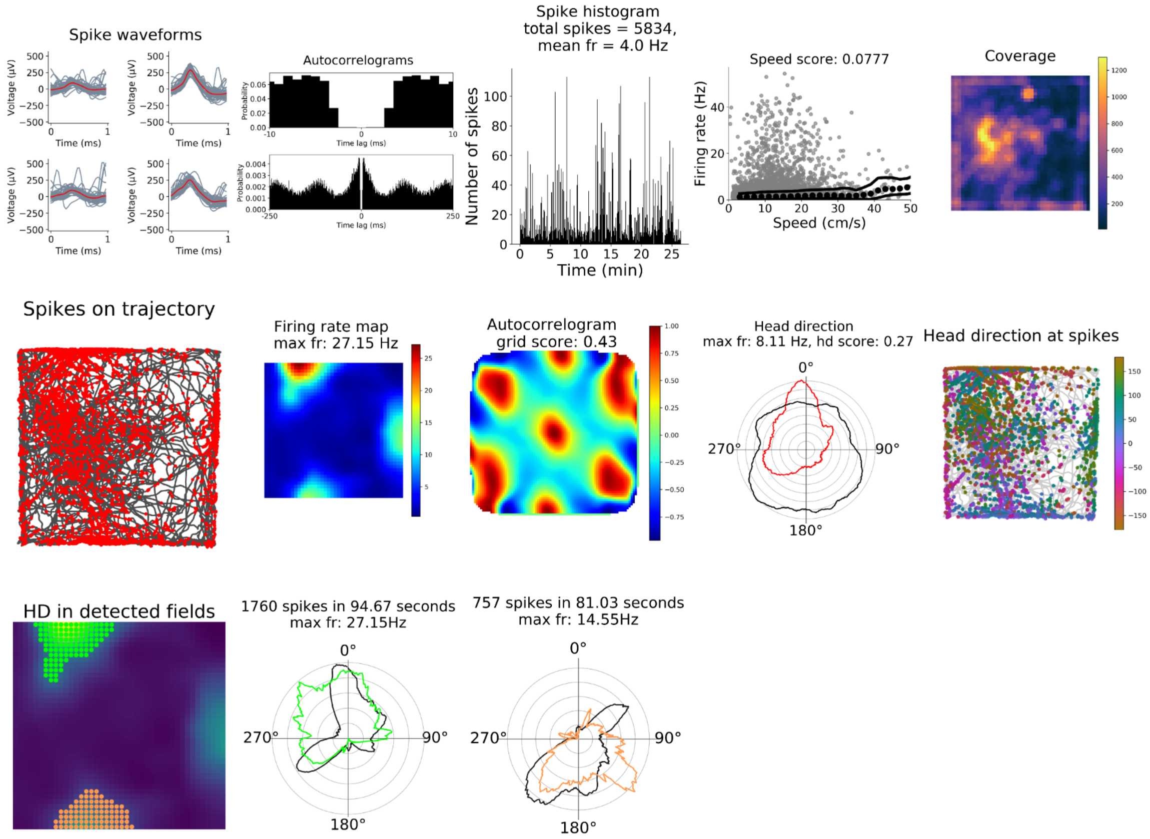
Spike waveforms
Voltage (µV)
Time (ms)
Autocorrelograms
Probability
Time lag (ms)
Spike histogram
total spikes = 5834,
mean fr = 4.0 Hz
Number of spikes
Time (min)
Speed score: 0.0777
Firing rate (Hz)
Speed (cm/s)
Coverage
Spikes on trajectory
Firing rate map
max fr: 27.15 Hz
Autocorrelogram
grid score: 0.43
Head direction
max fr: 8.11 Hz, hd score: 0.27
Head direction at spikes
HD in detected fields
1760 spikes in 94.67 seconds
max fr: 27.15Hz
757 spikes in 81.03 seconds
max fr: 14.55Hz

Animal ID: F

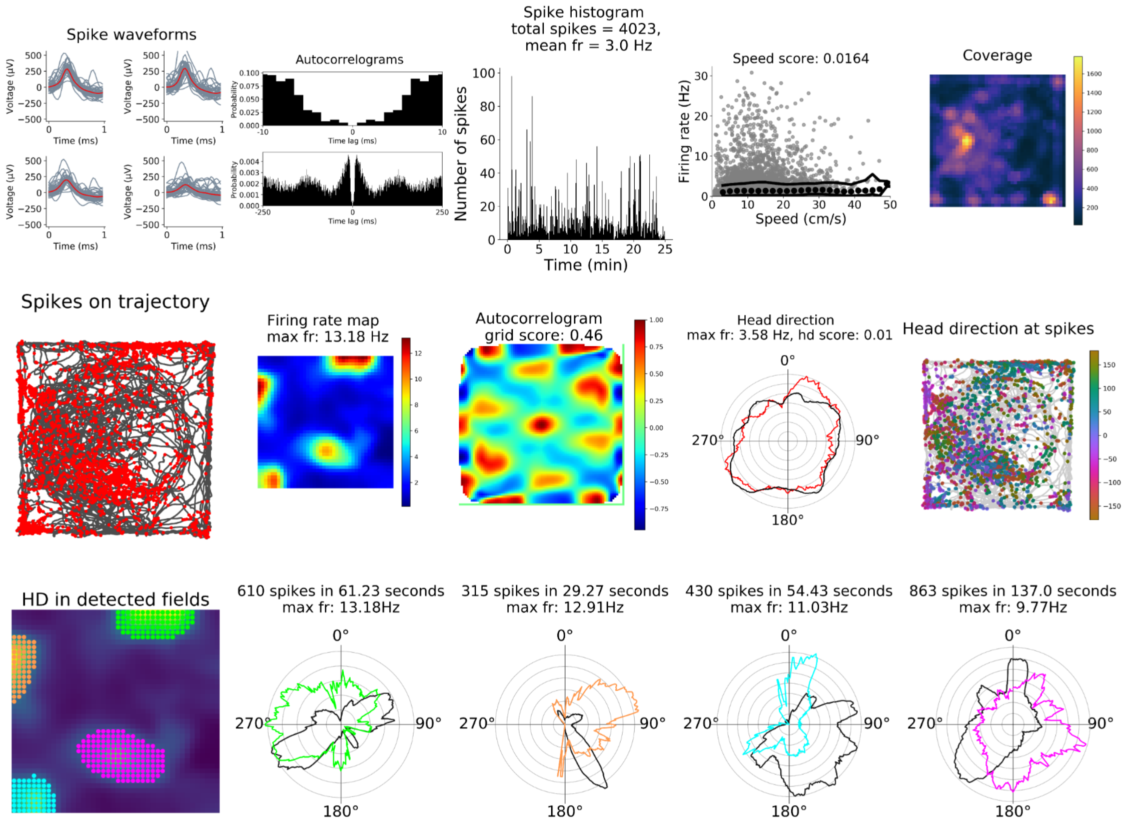
Spike waveforms
Autocorrelograms
Spike histogram
total spikes = 4023,
mean fr = 3.0 Hz
Number of spikes
Time (min)
Speed score: 0.0164
Firing rate (Hz)
Speed (cm/s)
Coverage
Spikes on trajectory
Firing rate map
max fr: 13.18 Hz
Autocorrelogram
grid score: 0.46
Head direction
max fr: 3.58 Hz, hd score: 0.01
Head direction at spikes
HD in detected fields
610 spikes in 61.23 seconds
max fr: 13.18Hz
315 spikes in 29.27 seconds
max fr: 12.91Hz
430 spikes in 54.43 seconds
max fr: 11.03Hz
863 spikes in 137.0 seconds
max fr: 9.77Hz

Animal ID: F

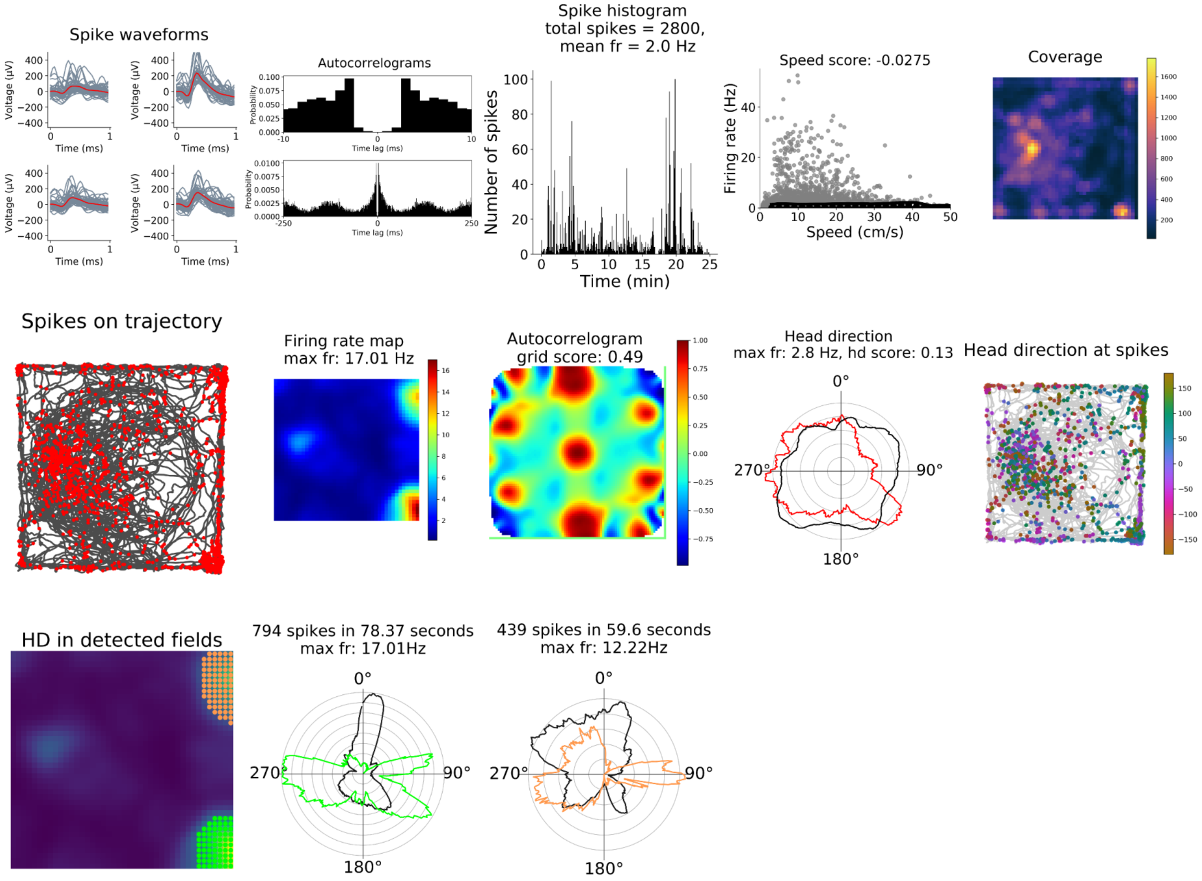

Spike waveforms
Autocorrelograms
Spike histogram
total spikes = 2800,
mean fr = 2.0 Hz
Speed score: -0.0275
Coverage
Spikes on trajectory
Firing rate map
max fr: 17.01 Hz
Autocorrelogram
grid score: 0.49
Head direction
max fr: 2.8 Hz, hd score: 0.13
Head direction at spikes
HD in detected fields
794 spikes in 78.37 seconds
max fr: 17.01Hz
439 spikes in 59.6 seconds
max fr: 12.22Hz
Time (ms)
Voltage (μV)
Time lag (ms)
Probability
Number of spikes
Time (min)
Firing rate (Hz)
Speed (cm/s)

Animal ID: F

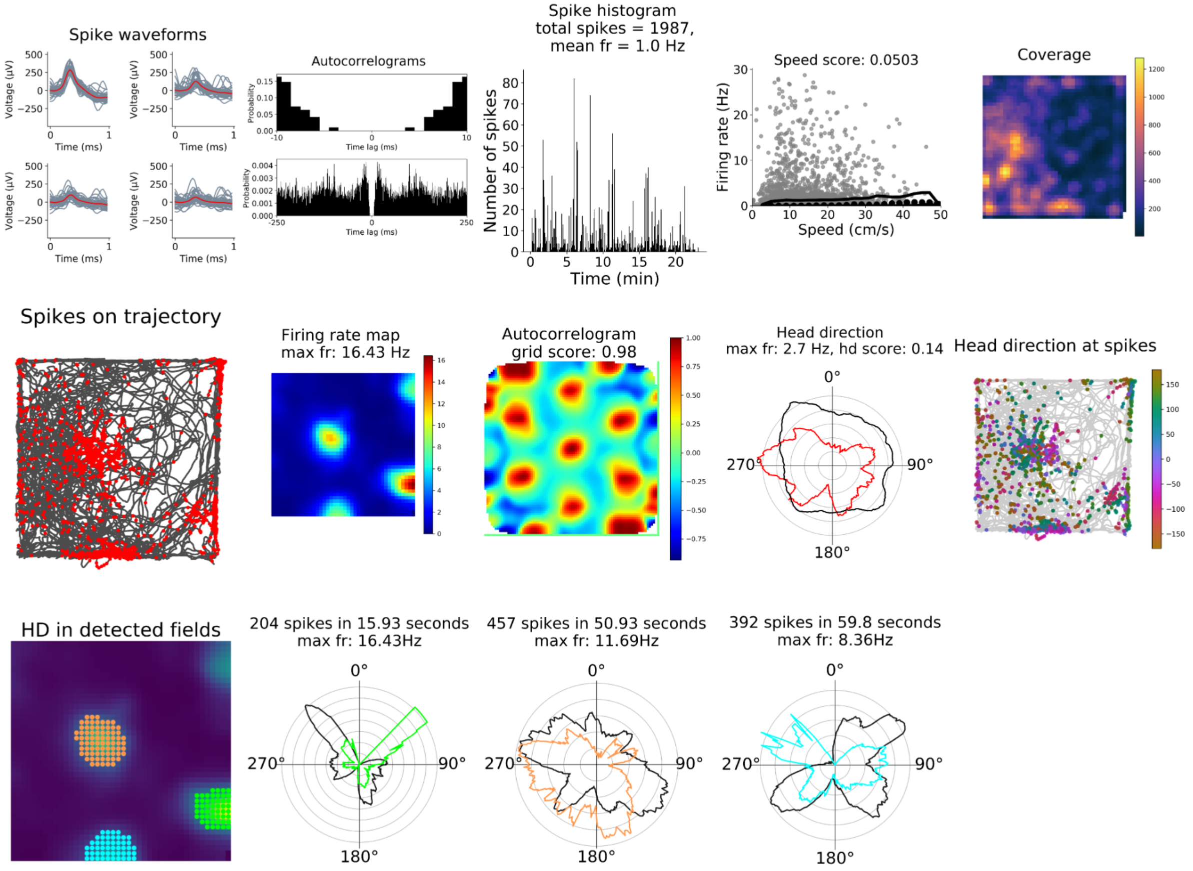
Spike waveforms
Voltage (µV)
Time (ms)
Autocorrelograms
Probability
Time lag (ms)
Spike histogram
total spikes = 1987,
mean fr = 1.0 Hz
Number of spikes
Time (min)
Speed score: 0.0503
Firing rate (Hz)
Speed (cm/s)
Coverage
Spikes on trajectory
Firing rate map
max fr: 16.43 Hz
Autocorrelogram
grid score: 0.98
Head direction
max fr: 2.7 Hz, hd score: 0.14
Head direction at spikes
HD in detected fields
204 spikes in 15.93 seconds
max fr: 16.43Hz
457 spikes in 50.93 seconds
max fr: 11.69Hz
392 spikes in 59.8 seconds
max fr: 8.36Hz
0°
90°
180°
270°

Animal ID: F

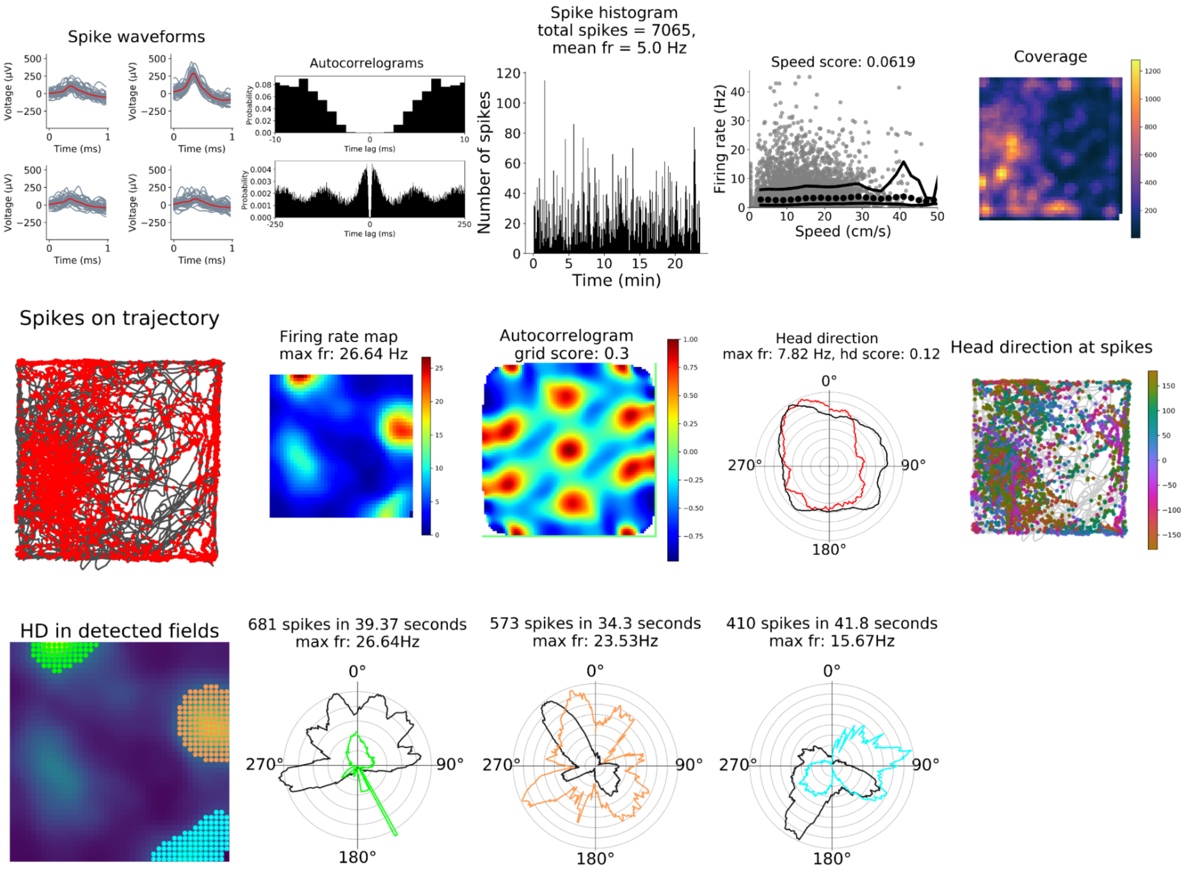

Spike waveforms
Autocorrelograms
Spike histogram
total spikes = 7065,
mean fr = 5.0 Hz
Speed score: 0.0619
Coverage
Spikes on trajectory
Firing rate map
max fr: 26.64 Hz
Autocorrelogram
grid score: 0.3
Head direction
max fr: 7.82 Hz, hd score: 0.12
Head direction at spikes
HD in detected fields
681 spikes in 39.37 seconds
max fr: 26.64Hz
573 spikes in 34.3 seconds
max fr: 23.53Hz
410 spikes in 41.8 seconds
max fr: 15.67Hz

Animal ID: F

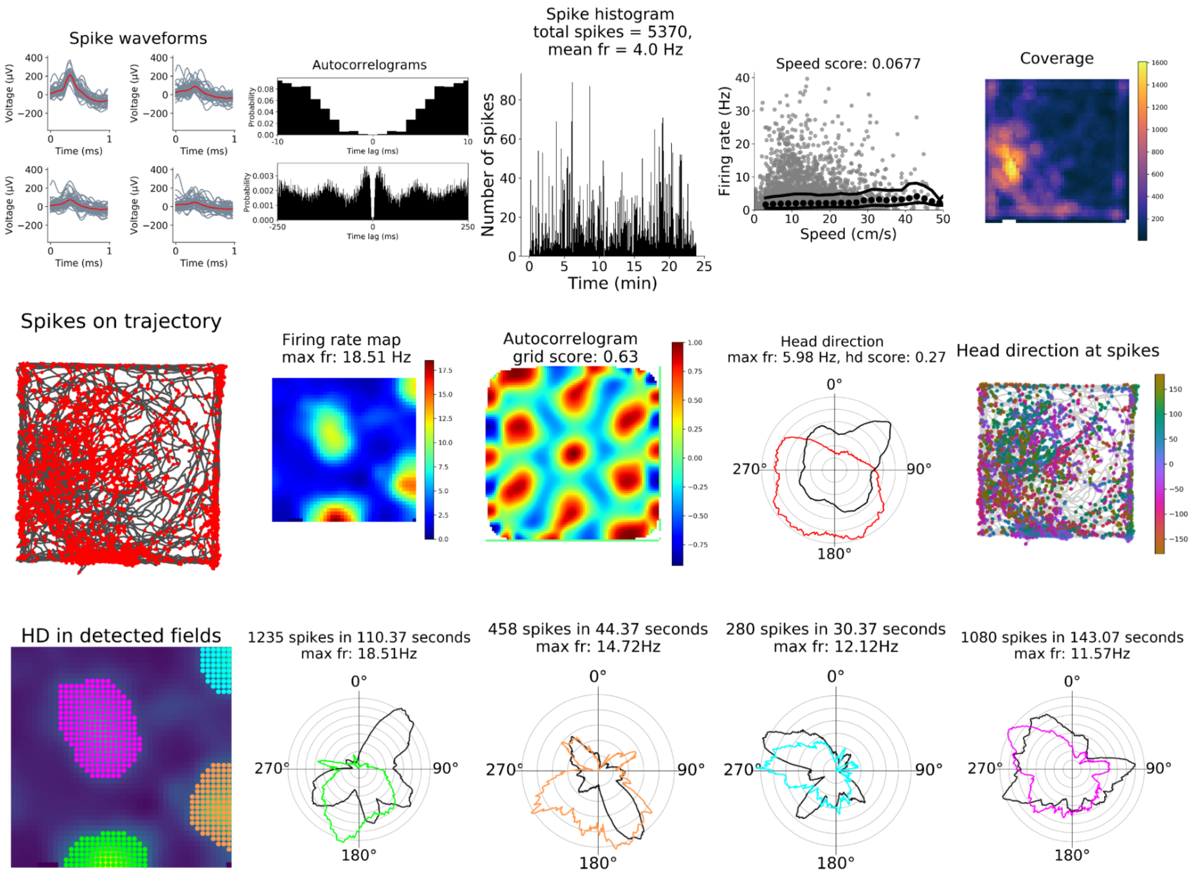
Spike waveforms
Autocorrelograms
Spike histogram
total spikes = 5370,
mean fr = 4.0 Hz
Number of spikes
Time (min)
Speed score: 0.0677
Firing rate (Hz)
Speed (cm/s)
Coverage
Spikes on trajectory
Firing rate map
max fr: 18.51 Hz
Autocorrelogram
grid score: 0.63
Head direction
max fr: 5.98 Hz, hd score: 0.27
Head direction at spikes
HD in detected fields
1235 spikes in 110.37 seconds
max fr: 18.51Hz
458 spikes in 44.37 seconds
max fr: 14.72Hz
280 spikes in 30.37 seconds
max fr: 12.12Hz
1080 spikes in 143.07 seconds
max fr: 11.57Hz

Animal ID: F

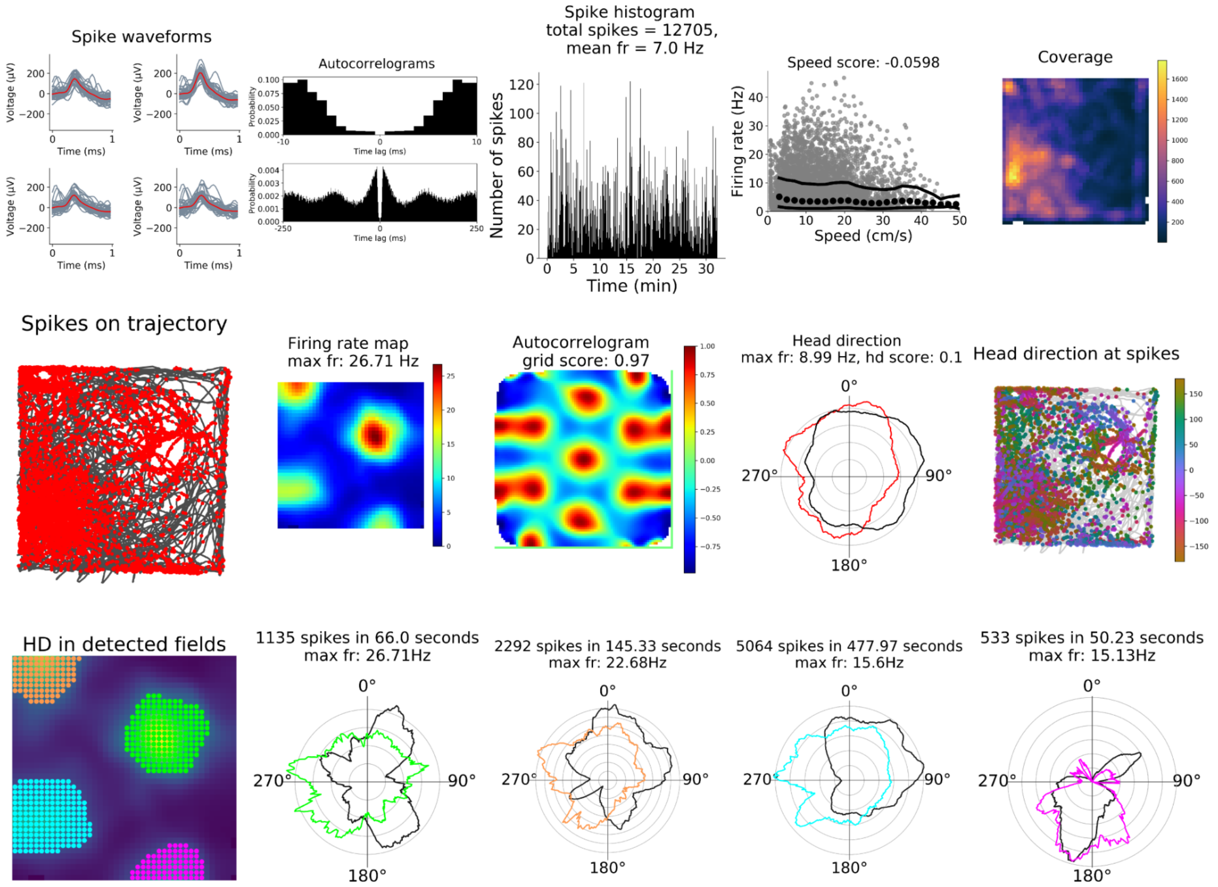
Spike waveforms
Voltage (µV)
Time (ms)
Autocorrelograms
Probability
Time lag (ms)
Spike histogram
total spikes = 12705,
mean fr = 7.0 Hz
Number of spikes
Time (min)
Speed score: -0.0598
Firing rate (Hz)
Speed (cm/s)
Coverage
Spikes on trajectory
Firing rate map
max fr: 26.71 Hz
Autocorrelogram
grid score: 0.97
Head direction
max fr: 8.99 Hz, hd score: 0.1
Head direction at spikes
HD in detected fields
1135 spikes in 66.0 seconds
max fr: 26.71Hz
2292 spikes in 145.33 seconds
max fr: 22.68Hz
5064 spikes in 477.97 seconds
max fr: 15.6Hz
533 spikes in 50.23 seconds
max fr: 15.13Hz
0°
90°
180°
270°

Animal ID: K

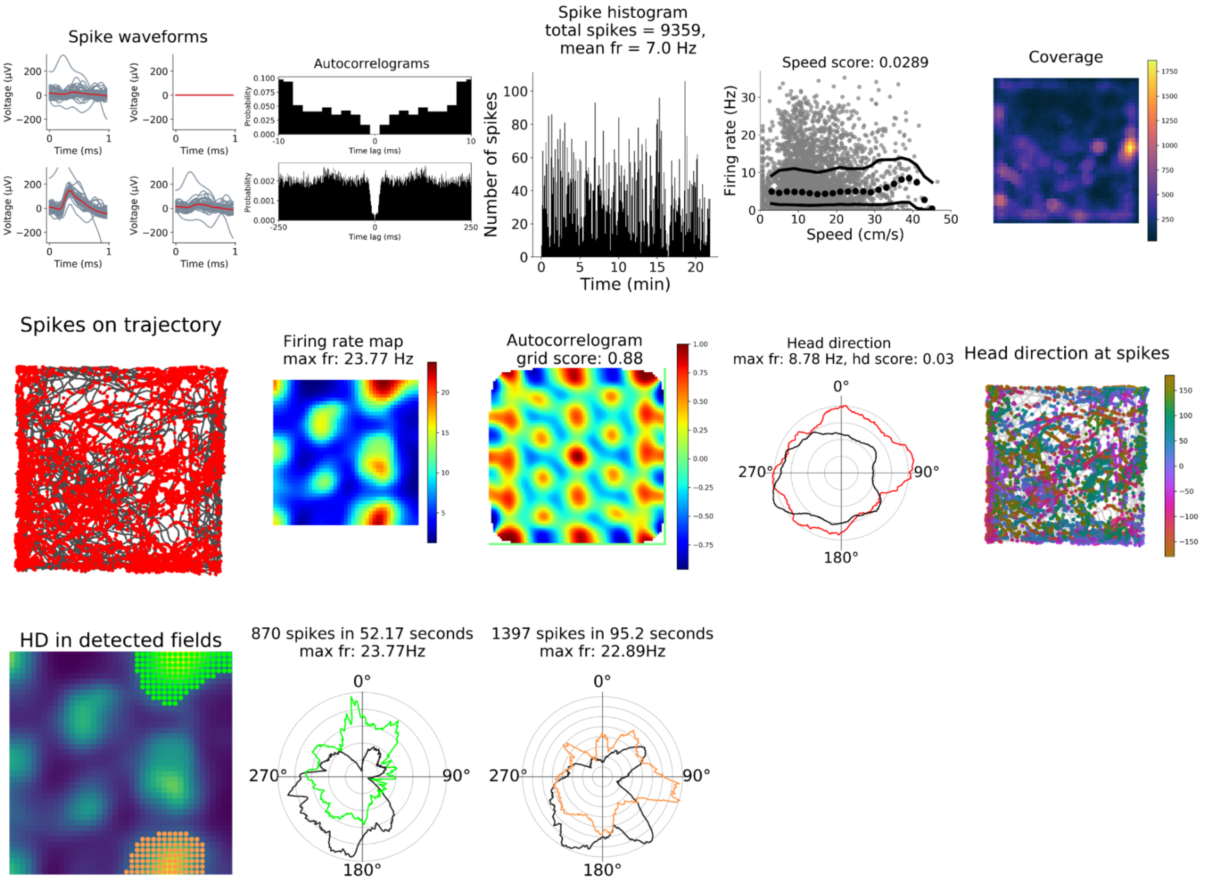
Spike waveforms
Voltage (µV)
Time (ms)
Autocorrelograms
Probability
Time lag (ms)
Spike histogram
total spikes = 9359,
mean fr = 7.0 Hz
Number of spikes
Time (min)
Speed score: 0.0289
Firing rate (Hz)
Speed (cm/s)
Coverage
Spikes on trajectory
Firing rate map
max fr: 23.77 Hz
Autocorrelogram
grid score: 0.88
Head direction
max fr: 8.78 Hz, hd score: 0.03
Head direction at spikes
HD in detected fields
870 spikes in 52.17 seconds
max fr: 23.77Hz
1397 spikes in 95.2 seconds
max fr: 22.89Hz
0°
90°
180°
270°

Animal ID: K

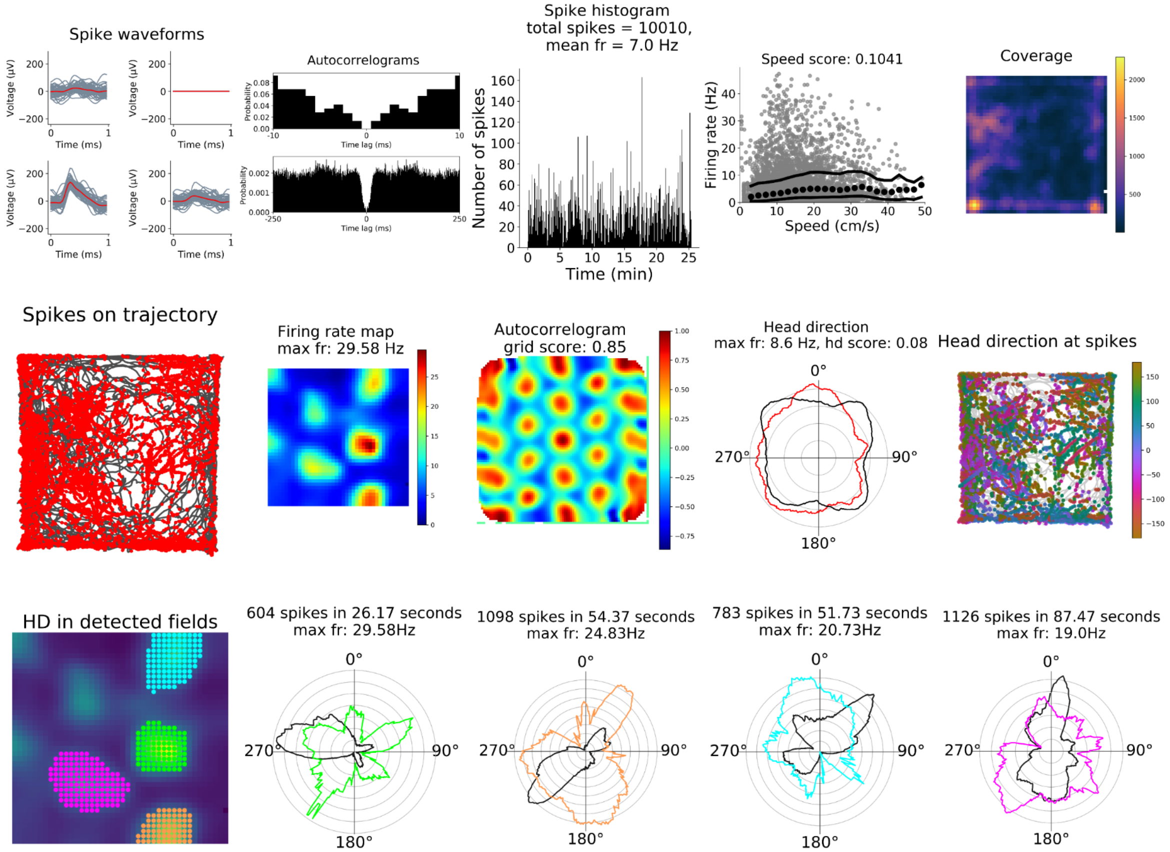
Spike waveforms
Voltage (µV)
Time (ms)
Autocorrelograms
Probability
Time lag (ms)
Spike histogram
total spikes = 10010,
mean fr = 7.0 Hz
Number of spikes
Time (min)
Speed score: 0.1041
Firing rate (Hz)
Speed (cm/s)
Coverage
Spikes on trajectory
Firing rate map
max fr: 29.58 Hz
Autocorrelogram
grid score: 0.85
Head direction
max fr: 8.6 Hz, hd score: 0.08
0°
90°
180°
270°
Head direction at spikes
HD in detected fields
604 spikes in 26.17 seconds
max fr: 29.58Hz
1098 spikes in 54.37 seconds
max fr: 24.83Hz
783 spikes in 51.73 seconds
max fr: 20.73Hz
1126 spikes in 87.47 seconds
max fr: 19.0Hz

Animal ID: K

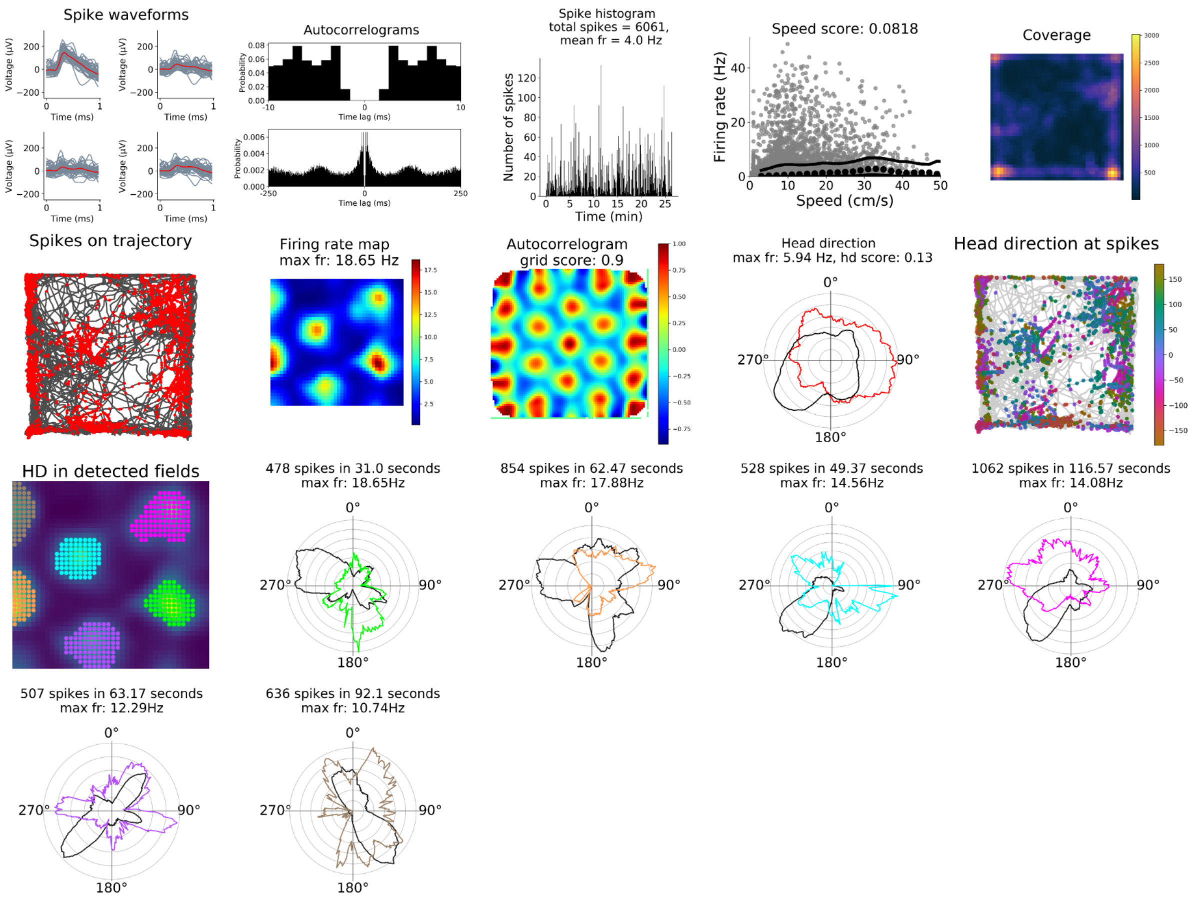

Spike waveforms
Voltage (µV)
Time (ms)
Autocorrelograms
Probability
Time lag (ms)
Spike histogram
total spikes = 6061,
mean fr = 4.0 Hz
Number of spikes
Time (min)
Speed score: 0.0818
Firing rate (Hz)
Speed (cm/s)
Coverage
Spikes on trajectory
Firing rate map
max fr: 18.65 Hz
Autocorrelogram
grid score: 0.9
Head direction
max fr: 5.94 Hz, hd score: 0.13
Head direction at spikes
HD in detected fields
478 spikes in 31.0 seconds
max fr: 18.65Hz
854 spikes in 62.47 seconds
max fr: 17.88Hz
528 spikes in 49.37 seconds
max fr: 14.56Hz
1062 spikes in 116.57 seconds
max fr: 14.08Hz
507 spikes in 63.17 seconds
max fr: 12.29Hz
636 spikes in 92.1 seconds
max fr: 10.74Hz
0°
90°
180°
270°

Animal ID: L

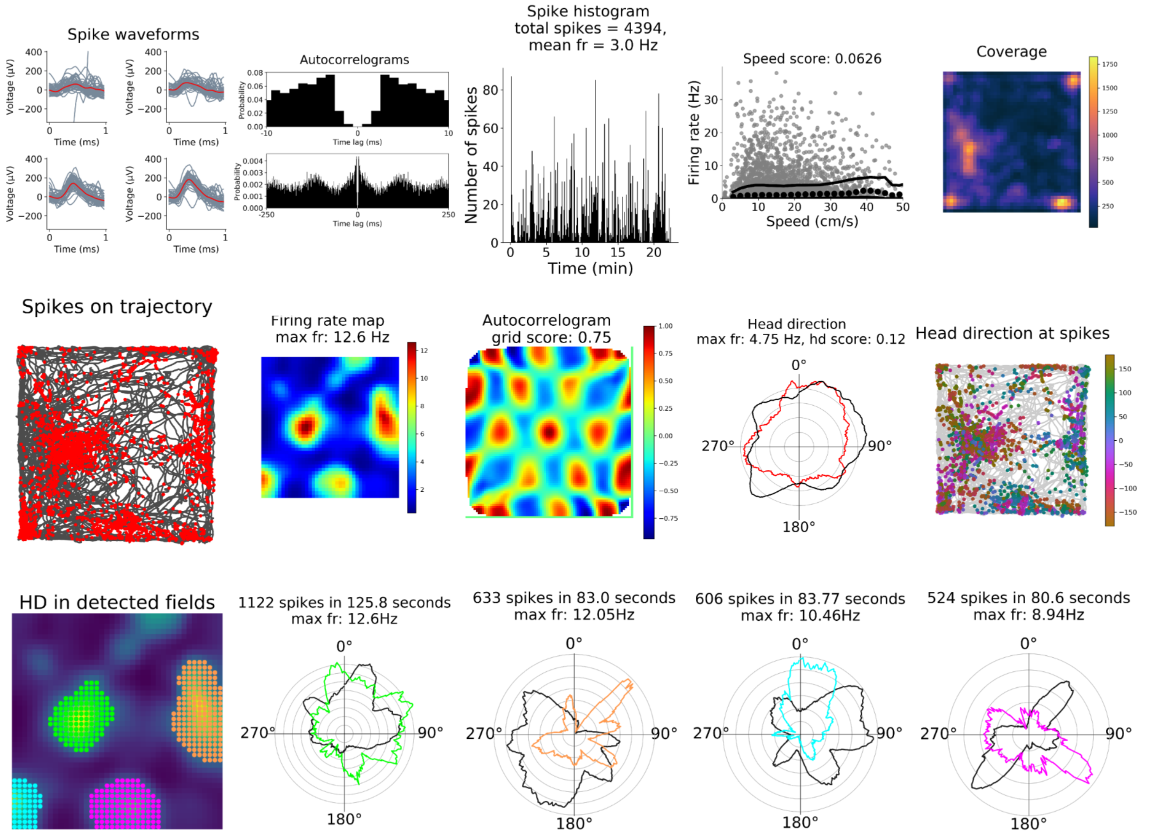

Spike waveforms
Autocorrelograms
Spike histogram
total spikes = 4394,
mean fr = 3.0 Hz
Speed score: 0.0626
Coverage
Spikes on trajectory
Firing rate map
max fr: 12.6 Hz
Autocorrelogram
grid score: 0.75
Head direction
max fr: 4.75 Hz, hd score: 0.12
Head direction at spikes
HD in detected fields
1122 spikes in 125.8 seconds
max fr: 12.6Hz
633 spikes in 83.0 seconds
max fr: 12.05Hz
606 spikes in 83.77 seconds
max fr: 10.46Hz
524 spikes in 80.6 seconds
max fr: 8.94Hz

Animal ID: M

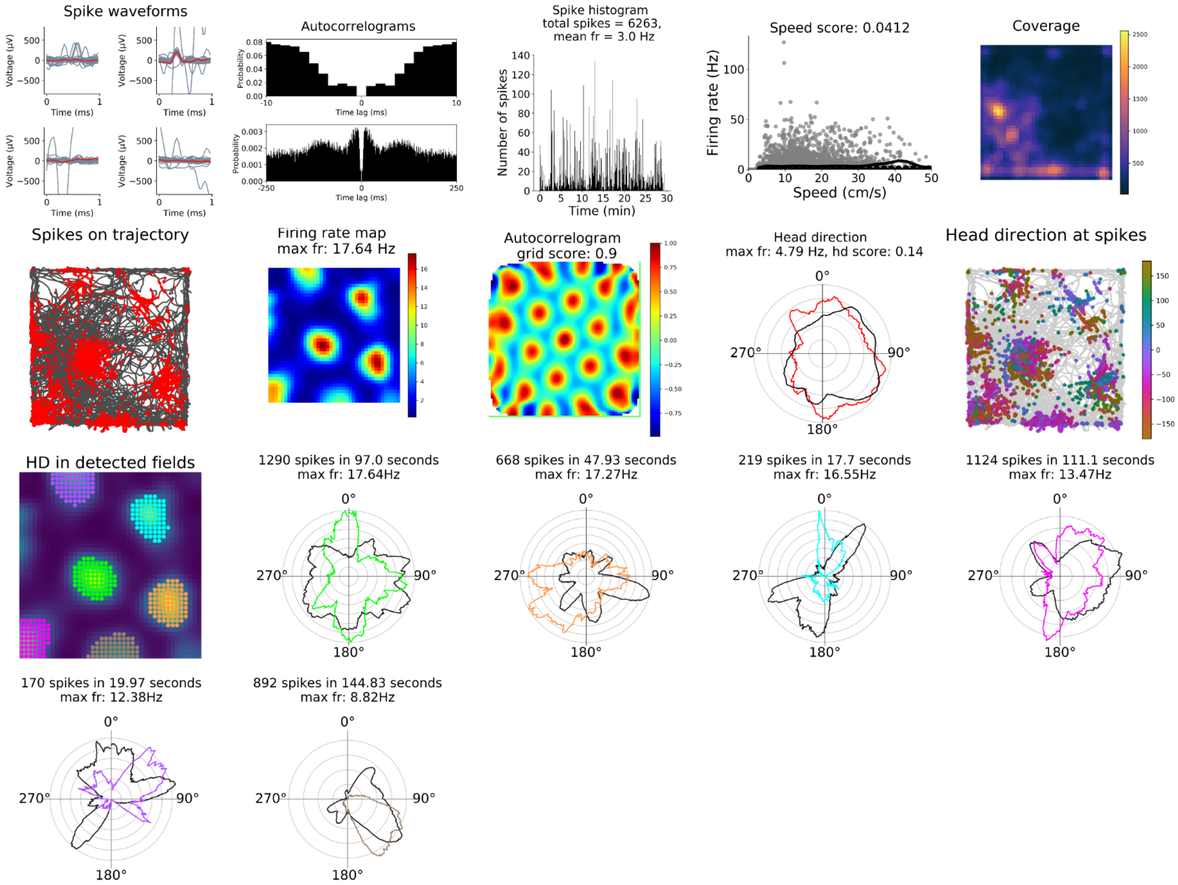
Spike waveforms
Autocorrelograms
Spike histogram
total spikes = 6263,
mean fr = 3.0 Hz
Speed score: 0.0412
Coverage
Spikes on trajectory
Firing rate map
max fr: 17.64 Hz
Autocorrelogram
grid score: 0.9
Head direction
max fr: 4.79 Hz, hd score: 0.14
Head direction at spikes
HD in detected fields
1290 spikes in 97.0 seconds
max fr: 17.64Hz
668 spikes in 47.93 seconds
max fr: 17.27Hz
219 spikes in 17.7 seconds
max fr: 16.55Hz
1124 spikes in 111.1 seconds
max fr: 13.47Hz
170 spikes in 19.97 seconds
max fr: 12.38Hz
892 spikes in 144.83 seconds
max fr: 8.82Hz
