# Supplementary material for: Grid cells are modulated by local head direction
Source: Nat Commun. 2020 Aug 24;11:4228. doi: 10.1038/s41467-020-17500-1 (PMC7445272; doi:10.1038/s41467-020-17500-1)
Supplement: Supplementary file 3 — Reporting Summary [file 41467_2020_17500_MOESM3_ESM.pdf]

nature research

Corresponding author(s): Matthew F Nolan

Last updated by author(s): Jun 24, 2020

Reporting Summary

Nature Research wishes to improve the reproducibility of the work that we publish. This form provides structure for consistency and transparency in reporting. For further information on Nature Research policies, see our Editorial Policies and the Editorial Policy Checklist.

Statistics

For all statistical analyses, confirm that the following items are present in the figure legend, table legend, main text, or Methods section.

n/a	Confirmed	
☐	☒	The exact sample size (*n*) for each experimental group/condition, given as a discrete number and unit of measurement
☐	☒	A statement on whether measurements were taken from distinct samples or whether the same sample was measured repeatedly
☐	☒	The statistical test(s) used AND whether they are one- or two-sided<br>*Only common tests should be described solely by name; describe more complex techniques in the Methods section.*
☐	☒	A description of all covariates tested
☐	☒	A description of any assumptions or corrections, such as tests of normality and adjustment for multiple comparisons
☐	☒	A full description of the statistical parameters including central tendency (e.g. means) or other basic estimates (e.g. regression coefficient) AND variation (e.g. standard deviation) or associated estimates of uncertainty (e.g. confidence intervals)
☐	☒	For null hypothesis testing, the test statistic (e.g. F, t, r) with confidence intervals, effect sizes, degrees of freedom and P value noted<br>*Give P values as exact values whenever suitable.*
☒	☐	For Bayesian analysis, information on the choice of priors and Markov chain Monte Carlo settings
☐	☒	For hierarchical and complex designs, identification of the appropriate level for tests and full reporting of outcomes
☐	☒	Estimates of effect sizes (e.g. Cohen's *d*, Pearson's *r*), indicating how they were calculated

Our web collection on statistics for biologists contains articles on many of the points above.

Software and code

Policy information about availability of computer code

Data collection	Open Ephys was used to record electrophysiology data and Bonsai 2.3 was used for motion tracking.
Data analysis	Analyses were carried out using MountainSort 3 (v 0.11.5), Python (version 3.5.1 in Anaconda environment 4.0) and R version: 3.3.1 (2016-06-21). All custom code is available at https://github.com/MattNolanLab/grid_cell_analysis

For manuscripts utilizing custom algorithms or software that are central to the research but not yet described in published literature, software must be made available to editors and reviewers. We strongly encourage code deposition in a community repository (e.g. GitHub). See the Nature Research guidelines for submitting code & software for further information.

Data

Policy information about availability of data

All manuscripts must include a data availability statement. This statement should provide the following information, where applicable:

- Accession codes, unique identifiers, or web links for publicly available datasets
- A list of figures that have associated raw data
- A description of any restrictions on data availability

All data we acquired will be made available from the Nolan Lab repository on the University of Edinburgh's DataShare site (https://datashare.is.ed.ac.uk/handle/10283/777). The Sargolini et al data was downloaded from the Kavli Institute's online database (https://www.ntnu.edu/kavli/research/grid-cell-data).

Field-specific reporting

Please select the one below that is the best fit for your research. If you are not sure, read the appropriate sections before making your selection.

[x] Life sciences   [ ] Behavioural & social sciences   [ ] Ecological, evolutionary & environmental sciences

For a reference copy of the document with all sections, see nature.com/documents/nr-reporting-summary-flat.pdf

Life sciences study design

All studies must disclose on these points even when the disclosure is negative.

Sample size	Power calculations were not performed. We chose the number of animals based on a similar study by Sargolini et al. (2006) that investigated directional firing of grid cells.
Data exclusions	We excluded from all analyses an animal that had to be terminated for health reasons. We only include mice in the grid cell analysis where we successfully recorded grid cells. The mice with grid cells were included in all analyses.
Replication	We used a publicly available data set from the Moser lab to repeat our analyses. All replication attempts were successful.
Randomization	There were no experimental groups in this study.
Blinding	We did not have multiple groups in the study. We used blinding for the histology analysis (blinding to animal identity when identifying tetrode locations).

Reporting for specific materials, systems and methods

We require information from authors about some types of materials, experimental systems and methods used in many studies. Here, indicate whether each material, system or method listed is relevant to your study. If you are not sure if a list item applies to your research, read the appropriate section before selecting a response.

Materials & experimental systems

n/a	Involved in the study	
[ ]	[x]	Antibodies
[x]	[ ]	Eukaryotic cell lines
[x]	[ ]	Palaeontology and archaeology
[ ]	[x]	Animals and other organisms
[x]	[ ]	Human research participants
[x]	[ ]	Clinical data
[x]	[ ]	Dual use research of concern

Methods

n/a	Involved in the study	
[x]	[ ]	ChIP-seq
[x]	[ ]	Flow cytometry
[x]	[ ]	MRI-based neuroimaging

Antibodies

Antibodies used	Rat mCherry (Thermo Fisher M11217), Goat anti-rat Alexa 555 (Thermo-Fisher A-21434)
Validation	Rat mCherry: According to the Thermo Fisher website, there are 16 publications using this antibody for IF. The product was tested on transgenic mice.<br>https://www.thermofisher.com/antibody/product/mCherry-Antibody-clone-16D7-Monoclonal/M11217

Animals and other organisms

Policy information about studies involving animals; ARRIVE guidelines recommended for reporting animal research

Laboratory animals	C57BJ mcos, p038. We report data from 8 mice (4 males and 4 females) from a total of 16 that were implanted with recording electrodes.<br><br>The age of the mice when tetrodes were implanted was 7 - 13 weeks (mean = 10.6 ± 1.7 weeks).
Wild animals	The study did not involve wild animals.
Field-collected samples	The study did not involve samples collected from the field.
Ethics oversight	All animal procedures were performed under a UK Home Office project license (PC198F2A0) in accordance with The University of Edinburgh Animal Welfare committee's guidelines. All procedures complied with the Animals (Scientific Procedures) Act, 1986, and

were approved by the Named Veterinary Surgeon and local ethical review committee.

Note that full information on the approval of the study protocol must also be provided in the manuscript.
